# Nitric oxide precursor interventions for post-exercise delayed onset muscle soreness: a systematic review and network meta-analysis of randomized placebo-controlled trials

**DOI:** 10.3389/fnut.2026.1868555

**Published:** 2026-10-15

**Authors:** Kai-Xuan Xu, Shuai Guo, Zhen-Fan Xie, Yu-Wei Xiao

**Affiliations:** 1 Department of Physical Education, Dankook University, Yongin-si, Gyeonggi-do, Republic of Korea; 2 School of Sports Science and Engineering, Hunan Institute of Engineering, Xiangtan, Hunan, China; 3 Department of Physical Education, Xiangtan University, Xiangtan, Hunan, China

**Keywords:** beetroot, citrulline, delayed onset muscle soreness, network meta-analysis, nitric oxide precursors, watermelon

## Abstract

**Background:**

Delayed onset muscle soreness (DOMS) commonly follows high-intensity or unaccustomed exercise. Nitric oxide (NO) precursors may reduce DOMS, but their effects remain uncertain. We conducted a systematic review and network meta-analysis to evaluate NO precursor interventions across recovery time points.

**Methods:**

PubMed, Embase, Web of Science, and CENTRAL were searched to February 2026 for randomized controlled trials in healthy adults. Interventions were classified into four nodes: beetroot-derived nitrate, watermelon-derived citrulline, synthetic or isolated amino acid precursors (L-arginine and citrulline malate), and watermelon plus added citrulline. Random-effects network meta-analyses were conducted separately at 24, 48, and 72 h. Effects were expressed as standardized mean differences (SMDs) with 95% confidence intervals (CIs); SUCRA rankings were exploratory. Risk of bias and evidence certainty were assessed using RoB 2 and CINeMA, with subgroup and sensitivity analyses examining robustness.

**Results:**

Nineteen trials involving 356 participants were included. At 24 h, watermelon plus added citrulline (SMD = −0.97, 95% CI −1.64 to −0.31) and beetroot-derived nitrate (SMD = −0.67, 95% CI −1.14 to −0.20) significantly reduced DOMS vs. placebo. At 48 h, beetroot-derived nitrate was the only intervention with a significant benefit (SMD = −1.18, 95% CI −1.91 to −0.45). No active intervention differed significantly from placebo at 72 h, while evidence for differences between active interventions was limited, with only watermelon plus added citrulline showing a significant advantage over synthetic or isolated amino acid precursors at 24 h. Beetroot-derived nitrate significantly reduced DOMS vs. placebo in the trained subgroup but not in the untrained subgroup. Sensitivity analyses generally supported the findings, although the beetroot-derived nitrate estimate was sensitive to some restrictions related to the exercise-induced muscle damage model.

**Conclusion:**

NO precursor supplementation may reduce post-exercise DOMS at specific recovery time points, particularly with beetroot-derived nitrate and watermelon plus added citrulline. However, evidence for differences between active interventions remains limited, and the sparse network, substantial heterogeneity, and low certainty of evidence preclude firm clinical conclusions.

**Systematic review registration:**

https://www.crd.york.ac.uk/PROSPERO/view/CRD420261373938, identifier: CRD420261373938.

## Introduction

Delayed onset muscle soreness (DOMS) is a common problem after high-intensity or unfamiliar exercise, particularly eccentric exercise. It usually develops within 24–48 h after exercise and peaks at around 72 h (1, 2). Rather than being caused by lactate accumulation, DOMS is now understood to result mainly from exercise-induced muscle microdamage and the inflammatory response that follows (1, 3). Eccentric exercise can disrupt muscle structure, alter calcium balance, and trigger the release of inflammatory and oxidative molecules, leading to pain, stiffness, reduced strength, and limited joint movement (3–5). These effects can impair short-term performance, disrupt subsequent training, and increase the risk of further injury (6, 7). For this reason, safe and effective recovery strategies that do not compromise training adaptation have become an important focus in sports nutrition and rehabilitation research (8–10).

Among nutritional strategies, nitric oxide (NO) precursors have attracted growing interest because of their roles in regulating blood flow, supporting metabolic recovery, and promoting muscle repair (11, 12). These supplements can be broadly described according to their delivery form as synthetic or isolated precursors, such as L-arginine, L-citrulline, and citrulline malate; and whole-food-derived precursors, such as beetroot juice and watermelon juice (12). This distinction refers to how the precursor is delivered and does not imply mutually exclusive biological pathways. Synthetic NO precursors entered the sports nutrition market earlier and are widely used in pre-workout products. However, evidence for their effects on DOMS is inconsistent, with generally small benefits and several studies showing no significant improvements in soreness or muscle damage markers (13, 14). In contrast, whole-food-derived precursors have received increasing attention in recent years, reflecting broader advances in the application of natural food ingredients and food science in sports nutrition (15). This trend is also consistent with the “food-first” approach promoted by the International Society of Sports Nutrition, which emphasizes obtaining relevant nutrients from whole foods whenever possible (16). In addition, studies of beetroot juice and watermelon juice suggest that these foods may reduce muscle soreness during the first 24–72 h after exercise and support functional recovery (17–19).

Although both types of intervention aim to increase NO availability, they may differ in both formulation and the pathways through which NO production is supported. Synthetic precursors such as L-arginine and L-citrulline primarily support NO production through the nitric oxide synthase pathway, whereas nitrate-containing interventions may increase NO availability through the nitrate–nitrite–NO pathway (12, 13). The latter pathway may remain active under certain hypoxic or acidic conditions, although its contribution to DOMS recovery remains uncertain. Synthetic or isolated precursors also usually provide a single active ingredient, whereas whole-food-derived interventions may additionally provide polyphenols, vitamins, and other plant compounds (20). These additional components may contribute to antioxidant and anti-inflammatory effects and could potentially support recovery through complementary mechanisms, although these mechanisms have not been directly established in the context of DOMS (20, 21).

Although previous systematic reviews and meta-analyses have examined NO precursors or nutrition-based strategies for DOMS, several limitations remain. First, whole-food-derived and synthetic NO precursors have often been grouped together as “NO boosters,” despite differences in formulation, precursor source, and broader nutritional composition (13, 20). Second, most reviews have focused on acute exercise performance rather than time-specific effects on post-exercise DOMS, particularly across the first 24–72 h of recovery (22, 23). Third, direct randomized comparisons between different NO precursors are limited, making it difficult for conventional pairwise meta-analysis to determine their relative effectiveness (24, 25). The common practice of combining acute and chronic supplementation protocols has further complicated interpretation (9, 13). Therefore, this study conducted a systematic review and network meta-analysis to evaluate distinct NO precursor intervention nodes defined according to supplement source/formulation and predominant NO-related precursor. The aim was to compare their relative effects on post-exercise DOMS across different recovery time points, and to provide clearer evidence to guide the use of NO precursors in exercise recovery (20, 21).

## Methods

This systematic review and network meta-analysis was conducted in accordance with PRISMA 2020 and the PRISMA-NMA extension to ensure clear and rigorous reporting throughout the study. To improve transparency, the review was registered with PROSPERO (CRD420261373938) (26, 27).

### Search strategy

We searched PubMed, Embase, Web of Science, and the Cochrane Central Register of Controlled Trials (CENTRAL) from inception to February 2026 for randomized controlled trials examining the effects of NO precursor interventions delivered either in food-based forms or as synthetic or isolated compounds on post-exercise delayed onset muscle soreness. Two reviewers independently performed the database search, screened titles and abstracts, and assessed full texts against predefined eligibility criteria. Disagreements were resolved by discussion, with input from a third reviewer when required.

To minimize the risk of missing relevant studies, we also manually screened ACSM and ECSS annual meeting abstracts, searched ClinicalTrials.gov, and checked the reference lists of included studies and relevant systematic reviews and meta-analyses. These sources were used only as supplementary checks to identify potentially eligible trials not captured by the database searches, and studies were included only when sufficient full-text data were available for extraction. The database search strategies are presented in Appendix 1.

### Eligibility criteria

We included full-text randomized controlled trials that examined the effects of eligible NO precursor interventions on delayed onset muscle soreness after exercise. Participants were healthy adults aged ≥18 years, with no prespecified upper age limit, who completed an acute exercise protocol designed to induce EIMD and subsequent DOMS. Training status was classified according to the descriptions provided in the original studies. Participants described as trained or as athletes were classified as the trained group, whereas those described as untrained or recreationally active were classified as the untrained group. Studies involving clinical populations, animal models, or exercise protocols not intended to induce EIMD and DOMS were excluded.

Eligible interventions were operationally classified into four active intervention nodes according to the source and formulation of the supplement and its predominant NO-related precursor: (1) beetroot-derived nitrate interventions; (2) watermelon-derived citrulline interventions; (3) synthetic or isolated amino acid precursor interventions; and (4) watermelon plus added citrulline interventions. Placebo served as the reference node. For brevity, these intervention nodes are hereafter referred to as beetroot-derived nitrate, watermelon-derived citrulline, synthetic or isolated amino acid precursors, and watermelon plus added citrulline, respectively. L-arginine and citrulline malate were grouped within the synthetic or isolated amino acid precursor node because both are isolated amino acid-based precursors acting within the arginine–NO pathway. Given their differences in formulation and pharmacokinetics, robustness was examined in a sensitivity analysis restricted to citrulline malate. Only trials in which the target precursor was the main active ingredient and the intervention was clearly described were included. The comparator had to be a placebo, such as nitrate-depleted beetroot juice or an inert capsule. Studies without a placebo control, or those in which the independent effect of the nitric oxide precursor could not be isolated, were excluded. For multi-arm trials, only eligible intervention arms were included, while arms with additional active co-interventions that could not be evaluated independently were excluded.

Studies were required to report at least one DOMS-related outcome, assessed using validated subjective or objective measures of DOMS. Relevant assessment time points included baseline, immediately after exercise, and 24, 48 and 72 h post-exercise. Studies that reported exercise performance only, without any DOMS-related outcome, were not included.

Both parallel-group trials and crossover trials with a clearly defined washout period were eligible. We excluded non-randomized studies, observational studies, conference abstracts, reviews, and studies from which relevant data could not be extracted. No language restrictions were applied, provided that an English abstract was available.

### Screening process

All records identified through the database searches were imported into EndNote X9.3.3 (Clarivate Analytics, Philadelphia, PA, USA) for reference management and deduplication. Study selection was completed in three stages. First, two reviewers independently screened titles and abstracts and retained studies that were potentially eligible or could not be excluded on the basis of the available information. Second, the full texts of these articles were assessed against the predefined inclusion and exclusion criteria. Finally, studies meeting all eligibility criteria were included in the review. Any disagreements were resolved through discussion and, when necessary, by consultation with a third reviewer.

### Data extraction

The eligibility criteria and data extraction items were organized according to the Population, Intervention, Comparator, Outcomes, and Study design (PICOS) framework (28). Before data extraction, we developed a standardized extraction form to ensure consistency. Two reviewers independently extracted the data, and any disagreements were resolved through discussion, with a third reviewer consulted when necessary. The extracted information included study characteristics (publication year, country, study design, eligibility criteria, and sample size), participant characteristics (age, sex, and training status), the EIMD induction protocol, and details of the intervention and control conditions, including food source, active ingredient, dose, formulation, supplementation schedule, and type of control (Table S2.1 in Supplementary Appendix 2). All control conditions were placebo based, such as nitrate-depleted beetroot juice or inert capsules.

We also extracted outcome-related information, including assessment time points, follow-up duration, and the methods used to assess DOMS. The primary outcome was DOMS at 48 h after EIMD, while DOMS at 24 and 72 h were secondary outcomes. DOMS was assessed using validated subjective or objective instruments, including the visual analog scale (VAS), pressure pain threshold (PPT), Likert scales, and numerical rating scales. For eligible studies, means and standard deviations were collected wherever possible at baseline and at each post-exercise recovery time point. For the primary analysis, post-exercise final values at each recovery time point were used rather than change-from-baseline scores. For crossover trials, first-period data collected before crossover were used when explicitly reported and treated as parallel-group data. When first-period data were unavailable, data from the full crossover trial were extracted and analyzed using methods appropriate for the crossover design. When results were presented only in figures, numerical values were estimated using Engauge Digitizer (version 12.1). Trials that did not report at least one DOMS-related outcome were excluded from the analysis.

### Quality assessment of evidence

Methodological quality was assessed using the Cochrane Risk of Bias 2.0 tool, which examines bias arising from the randomization process, departures from the intended intervention, missing outcome data, outcome measurement, and selective reporting (29). Two reviewers performed the assessments independently, and any disagreements were resolved through discussion. The certainty of the evidence was assessed using the CINeMA framework, which considers six domains: within-study bias, reporting bias, indirectness, imprecision, heterogeneity, and inconsistency (30, 31). To further examine the validity of the network, we also assessed the transitivity assumption by comparing the distribution of potential effect modifiers across studies contributing direct and indirect evidence.

### Statistical analysis

Network meta-analysis was performed in Stata 19.5 using the mvmeta command within a frequentist framework, with multivariate random-effects models applied throughout (32). Because DOMS changes over time, outcomes at 24, 48, and 72 h after exercise were analyzed separately. As studies used different instruments to assess DOMS, including VAS, PPT, Likert scales, and numerical rating scales, all continuous outcomes were expressed as standardized mean differences (SMDs) with 95% confidence intervals (CIs). Outcome directions were standardized before pooling so that lower values consistently represented less muscle soreness; accordingly, the direction of PPT-based effects was reversed. Negative SMDs therefore favored the intervention. For crossover trials without extractable first-period data, paired analyses were used when reported; otherwise, the variance was approximated using an imputed within-participant correlation coefficient, following established methods for incorporating crossover trials into meta-analyses (33, 34). Models were estimated using restricted maximum likelihood (35). A common heterogeneity variance (τ^2^) was assumed across all treatment comparisons, and the between-study correlation was fixed at 0.5 (36–38). Heterogeneity was interpreted as low (< 0.04), low to moderate (0.04–0.16), moderate to high (0.16–0.36), or high (>0.36) (37).

The evidence network at each time point was presented using network plots, while relative treatment effects were summarized in forest plots and league tables. Treatments were ranked using the surface under the cumulative ranking curve (SUCRA), with higher values indicating a greater probability of benefit for DOMS relief. SUCRA rankings were considered exploratory and interpreted alongside the corresponding effect estimates, particularly given the limited direct comparisons between active interventions. Network meta-analyses at each time point were based on a consistency model. Global inconsistency was assessed using the design-by-treatment interaction model, and local inconsistency was examined by node-splitting for the 24-and 48-h networks with closed loops. Node-splitting was not applicable to the 72-h network because no closed loop was present. Given the sparse network structure and limited power to detect inconsistency, the results were interpreted cautiously.

To test the robustness of the findings, sensitivity analyses were conducted to examine the influence of EIMD induction models, outcome assessment, precursor type, supplementation timing, formulation, and the assumed within-participant correlation coefficient for crossover trials. Studies were grouped into four categories: category A, laboratory-controlled lower-limb mechanical models; category B, real-world, sport-specific, or mixed endurance-based models; category C, resistance-based models involving mixed muscle groups or not confined to the lower limbs; and category D, non-typical models or those with less certain muscle-damaging effects. Sensitivity analyses were performed by excluding each EIMD model category in turn and by restricting the analysis to category A. Additional analyses excluded PPT-based outcomes, restricted the synthetic or isolated amino acid precursor node to citrulline malate interventions, restricted the analysis to pre-exercise supplementation protocols, excluded the powder-based beetroot formulation, and varied the imputed within-participant correlation coefficient from 0.50 in the primary analysis to 0.30, 0.70 and 0.90 in sensitivity analyses. These analyses were used to assess the potential influence of outcome measurement and within-node variation in precursor type, timing, and formulation.

## Results

### Literature selection

A total of 2,548 records were identified, including 579 from PubMed, 520 from the Cochrane Library, 1,169 from Embase, and 280 from Web of Science. No additional unique potentially eligible records were identified from the supplementary sources, and none entered the deduplication or screening process. After 1,379 duplicates were removed, 1,169 records remained for title and abstract screening. Of these, 1,086 were excluded because they were not relevant to the review question.

The full texts of 83 articles were then assessed for eligibility. Sixty-four studies were excluded for the following reasons: the intervention did not meet the inclusion criteria, or the independent effect of the plant-derived nitric oxide precursor could not be isolated from a combined intervention (*n* = 38); the exercise-induced muscle damage model was not eligible, or the study was not conducted in a post-exercise recovery setting (*n* = 7); outcome data were insufficient, not relevant, or could not be extracted (*n* = 11); or the full text was unavailable, or the data were not extractable (*n* = 8). In total, 19 studies met the inclusion criteria (20, 39–56) and were included in the quantitative synthesis and network meta-analysis (Figure 1).

**Figure 1:**
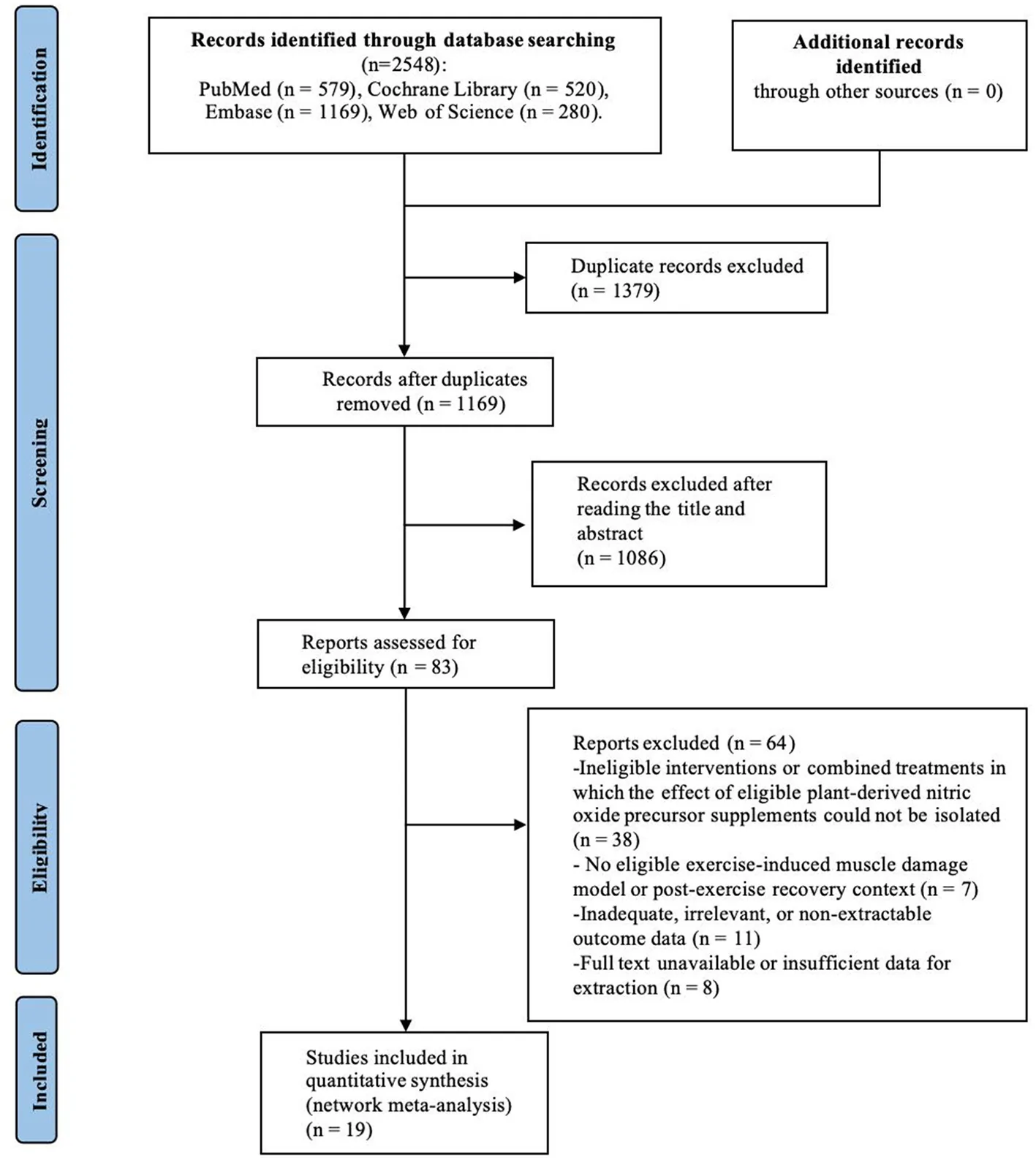
PRISMA flow diagram of study identification and selection process.

### Study characteristics

A total of 19 randomized controlled trials published between 2010 and 2025 were included, representing 356 participants. The studies were conducted in six countries, most commonly the United Kingdom, Iran, and Spain. Sample sizes ranged from 7 to 41 participants, with mean ages spanning approximately 21 to 42 years. Most studies enrolled predominantly male participants, and both trained and untrained populations were represented. The exercise-induced muscle damage protocols varied across studies and included eccentric jump exercise, repeated sprint exercise, resistance training, endurance or sport-specific exercise models, and high-altitude trekking. Four intervention nodes were identified. Beetroot-derived nitrate was the most frequently investigated node [10 studies (20, 39–45, 50, 51)], followed by synthetic or isolated amino acid precursors [5 studies (49, 53–56)], watermelon plus added citrulline [4 studies (46–48, 52)], and watermelon-derived citrulline [2 studies (46, 48)]. Two studies contributed to both watermelon-related nodes because they included separate watermelon-derived citrulline and watermelon plus added citrulline arms. Supplements were administered in the form of juices, beverages, or powders. Most were given either as acute supplementation before exercise or as multi-day supplementation, typically for 1 to 4 days, although a few studies extended the intervention to 7 days. Placebo was the main comparator across studies, including placebo juices, placebo beverages, cornstarch, and control water, and most treatment comparisons were connected through these placebo groups. The visual analog scale was the most commonly used outcome measure, although pressure pain threshold, Likert scales, and numerical rating scales were also reported. These measures were used primarily to assess DOMS at different post-exercise recovery time points (Table 1, Table S2.2 in Supplementary Appendix 2).

**Table 1:** Characteristics of the included studies.

References	Country	Design	Randomized treatments	Age (mean ±SD, yrs)	Sex (M/F)	Training Status	Intervention	Intervention node	Comparator	Duration
Salem et al. (20)	Tunisia	RCT	*n* = 11 Crossover	21.3 ± 1.9	11/0	untrained/recreational	Beetroot powder dissolved in juice	Beetroot-derived nitrate	Matched placebo juice	4 days
Ahmadpour et al. (39)	Iran	RCT	*n* = 10 Crossover	22 ± 3.6	10/0	trained/athlete	Beetroot juice	Beetroot-derived nitrate	Placebo juice	1 day
Clifford et al. (40)	UK	RCT	BT = 10 PLA = 10	BT = 22 ± 6 PLA = 21 ± 3	20/0	untrained/recreational	Beetroot juice	Beetroot-derived nitrate	Matched placebo juice	3 days
Clifford et al. (41)	UK	RCT	BTJ = 10 PLA=10	BTJ = 23 ± 3 PLA= 21 ± 2	20/0	trained/athlete	Beetroot juice	Beetroot-derived nitrate	Matched placebo juice	4 days
Clifford et al. (42)	UK	RCT	BTJ = 17 PLA=17	BTJ = 42 ± 10 PLA=39 ± 12	BTJ = 10/7 PLA = 11/6	trained/athlete	Beetroot beverage	Beetroot-derived nitrate	Matched placebo beverage	3 days
Clifford et al. (43)	UK	RCT	BTJ = 10 PLA=10	BTJ = 22.6 ± 2.8 PLA=21.0 ± 1.4	20/0	untrained/recreational	Beetroot juice	Beetroot-derived nitrate	Matched placebo juice	3 days
Hemmatinafar et al. (44)	Iran	RCT	*n* = 12 Crossover	26.0 ± 3.0	0/12	trained/athlete	Beetroot juice	Beetroot-derived nitrate	Matched placebo drink	2 days
Daneshparvar et al. (45)	Iran	RCT	BTJ = 9 PLA=9	37 ± 7	9/9	trained/athlete	Beetroot juice/beverage	Beetroot-derived nitrate	Matched placebo beverage	1 day
Tarazona-Díaz et al. (46)	Spain	RCT	*n* = 7 Crossover	22.7 ± 0.8	7/0	untrained/recreational	Natural and enriched watermelon juice	Watermelon-derived citrulline; Watermelon plus added citrulline	Matched fruit-flavored placebo juice	1 day
Martínez-Sánchez et al. (47)	Spain	RCT	*n* = 21 Crossover	35.3 ± 11.4	21/0	trained/athlete	Enriched watermelon juice	Watermelon plus added citrulline	Matched placebo juice	1 day
Martínez-Sánchez et al. (48)	Spain	RCT	*n* = 19 Crossover	23.9 ± 3.7	19/0	trained/athlete	Natural and enriched watermelon juice	Watermelon-derived citrulline; Watermelon plus added citrulline	Matched placebo	1 day
Pérez-Guisado et al. (49)	Spain	RCT	*n* = 41 Crossover	29.80 ± 7.64	41/0	untrained/recreational	Citrulline malate beverage	Synthetic or isolated amino acid precursors	Matched placebo beverage	1 day
Daab et al. (50)	Tunisia	RCT	*n* = 13 Crossover	22.12 ± 0.56	13/0	trained/athlete	Beetroot juice	Beetroot-derived nitrate	Matched placebo juice	7 days
Clifford et al. (51)	UK	RCT	H-BT = 10 PLA=10	21 ± 3	20/0	untrained/recreational	Beetroot concentrate	Beetroot-derived nitrate	Matched placebo juice	3 days
Blohm et al. (52)	USA	RCT	*n* = 27 Crossover	M: 24.8 ± 0.52 F: 24.4 ± 0.41	13/14	untrained/recreational	Fortified watermelon beverage	Watermelon plus added citrulline	Matched placebo juice	1 day
Andrade et al. (53)	Brazil	RCT	ARG =10 PLA = 10	ARG: 23.0 ± 4.0 PLA: 22.5 ± 3.3	14/6	untrained/recreational	L-arginine powder	Synthetic or isolated amino acid precursors	Cornstarch placebo	4 days
Chappell et al. (54)	UK	RCT	*n* = 19 Crossover	25.7 ± 7.7	12/7	untrained/recreational	Citrulline malate powder	Synthetic or isolated amino acid precursors	Citric acid placebo	1 day
Chappell et al. (55)	UK	RCT	*n* = 15 Crossover	23.67 ± 2.41	11/4	untrained/recreational	Citrulline malate powder	Synthetic or isolated amino acid precursors	Citric acid placebo	1 day
da Silva et al. (56)	Brazil	RCT	*n* = 9 Crossover	24.0 ± 3.3	9/0	untrained/recreational	Citrulline malate powder	Synthetic or isolated amino acid precursors	Cornstarch placebo	4 days

RCT, randomized controlled trial; SD, standard deviation; M, male; F, female; H-BT, high-dose beetroot juice; BTJ, beetroot juice; PLA, placebo; ARG, arginine group.

### Risk of bias, certainty of evidence, and consistency

Risk of bias was assessed using the Cochrane Risk of Bias 2 tool. Overall, the methodological quality of the included trials was good. Of the 19 studies, 16 (84.2%) were rated as low risk of bias, two (10.5%) as having some concerns, and one (5.3%) as high risk (Figure 2). The single high overall risk judgement reflected concerns in both the randomization process and deviations from the intended intervention. At the domain level, missing outcome data, outcome measurement, and selective reporting were consistently judged to be low risk, whereas the main potential sources of bias were the randomization process and deviations from the intended intervention (Table S3 in Supplementary Appendix 3). Global inconsistency was not statistically significant at 24, 48, or 72 h (all *p* > 0.05). In the 24- and 48-h networks, which contained closed loops, node-splitting also showed no significant differences between direct and indirect estimates, although the indirect estimates were imprecise because evidence within the loops was limited. Local inconsistency could not be assessed at 72 h because no closed loop was present. Between-study heterogeneity was moderate to high at 24 h (τ^2^ = 0.3585) and high at 48 h (τ^2^ = 1.0471) and 72 h (τ^2^ = 1.2337) (Tables S4.1–S4.3 in Supplementary Appendix 4). Transitivity assessment showed broadly comparable age distributions across intervention nodes, but differences in sex distribution, training status, EIMD models, supplementation regimens, and outcome measures introduced some uncertainty regarding indirect comparisons (Table S7.1 in Supplementary Appendix 7). For the primary 48 h DOMS outcome, CINeMA ratings were generally low, mainly because of imprecision and inconsistency, with risk-of-bias contributions and comparison-level certainty assessments presented in Supplementary Appendix 7 (Figures S7.1–S7.2 and Table S7.2). Egger's tests did not indicate statistically significant small-study effects at 24, 48, or 72 h, although these network-level results should be interpreted cautiously given the limited number of studies and the pooling of different active-vs.-placebo comparisons (Figures S8.1–S8.3 in Supplementary Appendix 8).

**Figure 2:**
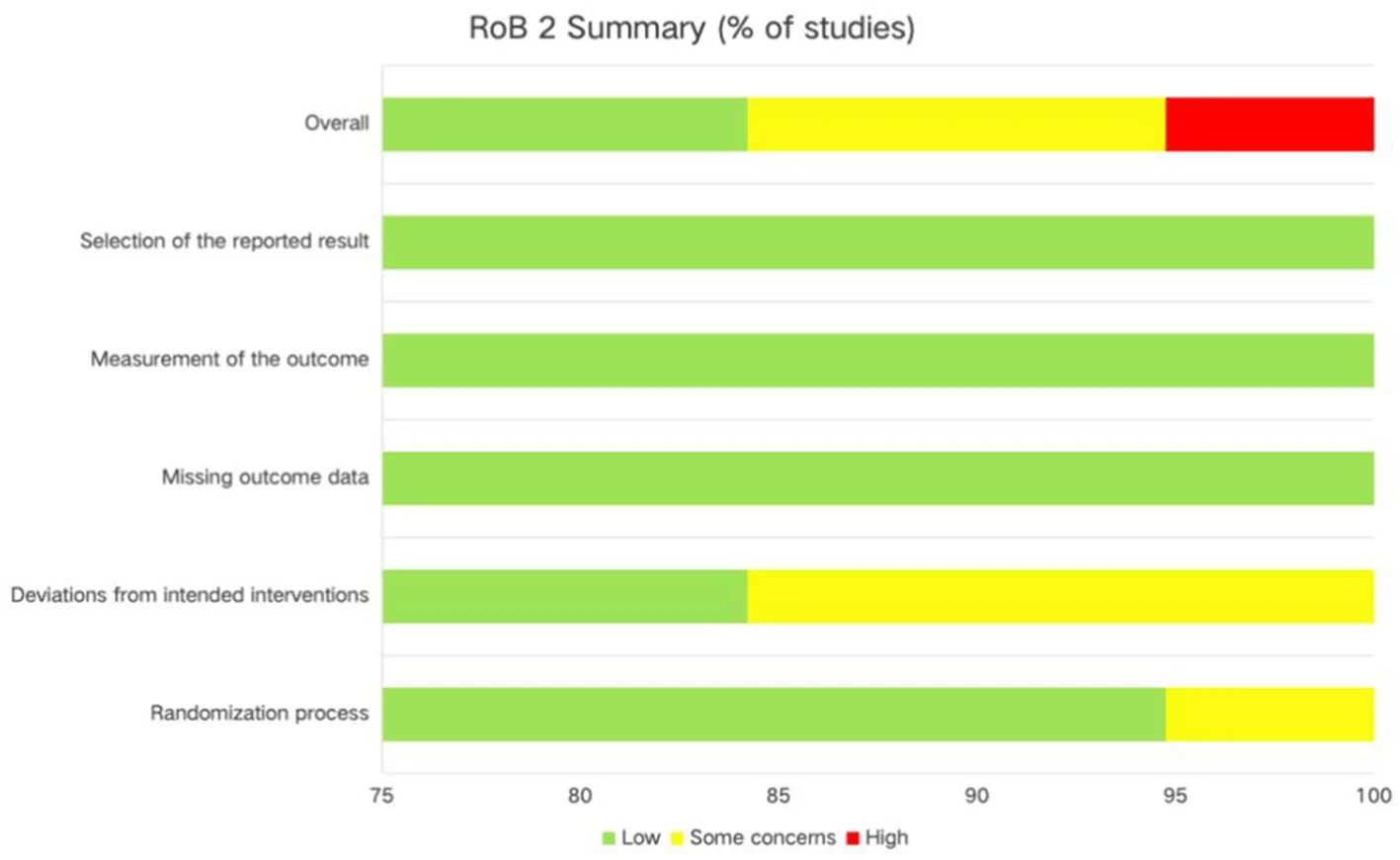
Risk-of-bias assessment of the included studies. Green indicates low risk of bias, yellow indicates some concerns, and red indicates high risk of bias.

### Primary network analysis

For the 48 h DOMS outcome, 18 studies involving 329 participants and five intervention nodes were included in the network meta-analysis. The network plot showed that placebo was the main common comparator, linking most active interventions, and the network was fully connected with no isolated nodes (Figure 3). Overall, all active interventions showed point estimates favoring a reduction in DOMS at 48 h compared with placebo, although the 95% CIs crossed the null for all interventions except beetroot-derived nitrate. Based on the pooled estimates and SUCRA rankings (Figure S5.1, Table S5.1 in Supplementary Appendix 5), beetroot-derived nitrate had the highest SUCRA ranking and was the only intervention associated with a statistically significant benefit over placebo (SMD = −1.18, 95% CI −1.91 to −0.45; SUCRA = 92.5%). The other interventions did not reach statistical significance, but the SUCRA rankings placed watermelon plus added citrulline second (58.8%), followed by synthetic or isolated amino acid precursors (36.1%) and watermelon-derived citrulline (35.5%), while placebo ranked last (27.1%). The league table was consistent with the main analysis, showing a significant benefit only for beetroot-derived nitrate over placebo, with no significant differences in the remaining pairwise comparisons (Table S6.1 in Supplementary Appendix 6). The certainty of the evidence for this outcome was rated as low by CINeMA, mainly because of imprecision and inconsistency in the network (Table S7.2 in Supplementary Appendix 7).

**Figure 3:**
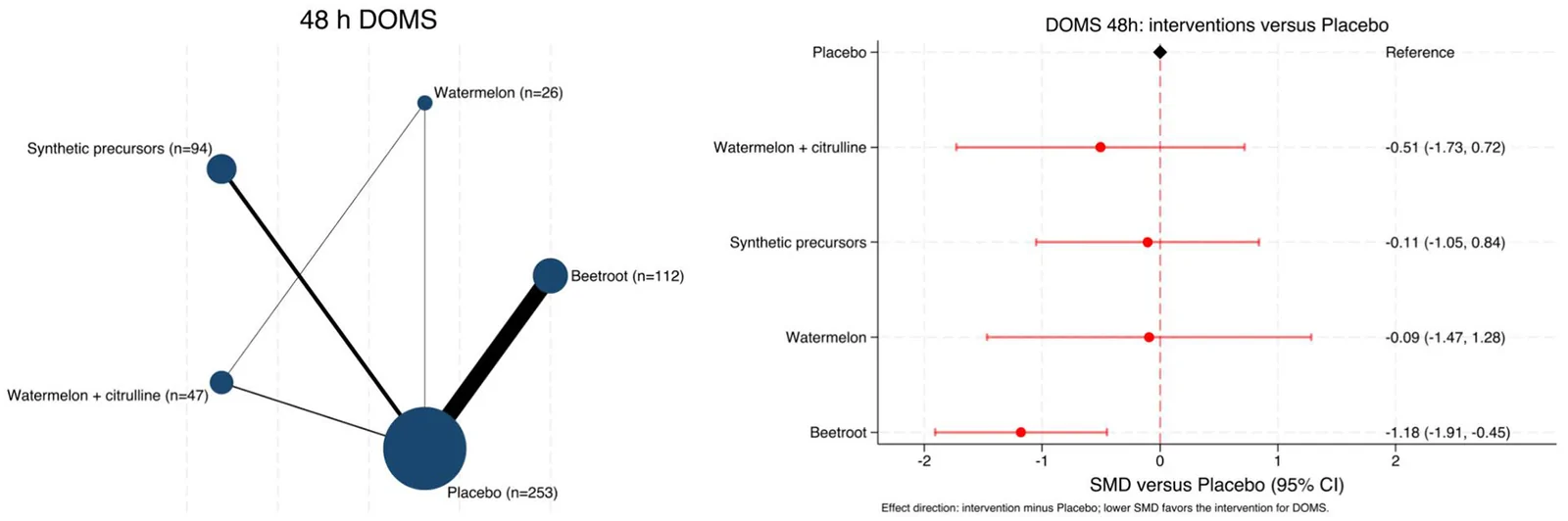
Network plot and forest plot for 48-h DOMS. Node labels: Beetroot, beetroot-derived nitrate; Watermelon, watermelon-derived citrulline; Synthetic precursors, synthetic or isolated amino acid precursors; Watermelon + citrulline, watermelon plus added citrulline. Placebo was the reference treatment.

### Secondary network analysis

For the 24 h DOMS outcome, the network meta-analysis included 19 studies with 356 participants across five intervention nodes. The network was fully connected, with placebo serving as the main common comparator and no isolated nodes identified (Figure 4). Overall, the active interventions tended to reduce DOMS at 24 h relative to placebo, although not all comparisons reached statistical significance (Figure 5A). Based on the pooled estimates and SUCRA rankings, watermelon plus added citrulline had the highest SUCRA ranking and showed a significant benefit over placebo (SMD = −0.97, 95% CI −1.64 to −0.31; SUCRA = 90.7%). Beetroot-derived nitrate ranked second and demonstrated a significant reduction in 24 h DOMS compared with placebo (SMD = −0.67, 95% CI −1.14 to −0.20; SUCRA = 70.9%). The remaining interventions were not significantly different from placebo, although watermelon-derived citrulline ranked third (SUCRA = 51.9%) and synthetic or isolated amino acid precursors ranked lower (SUCRA = 22.1%), while placebo ranked last (SUCRA = 14.4%) (Figure S5.2, Table S5.2 in Supplementary Appendix 5). The league table supported these findings, showing significant benefits over placebo for watermelon plus added citrulline and beetroot-derived nitrate. A significant difference was also observed between synthetic or isolated amino acid precursors and watermelon plus added citrulline, favoring the latter; no other pairwise comparisons were statistically significant (Table S6.2 in Supplementary Appendix 6).

**Figure 4:**
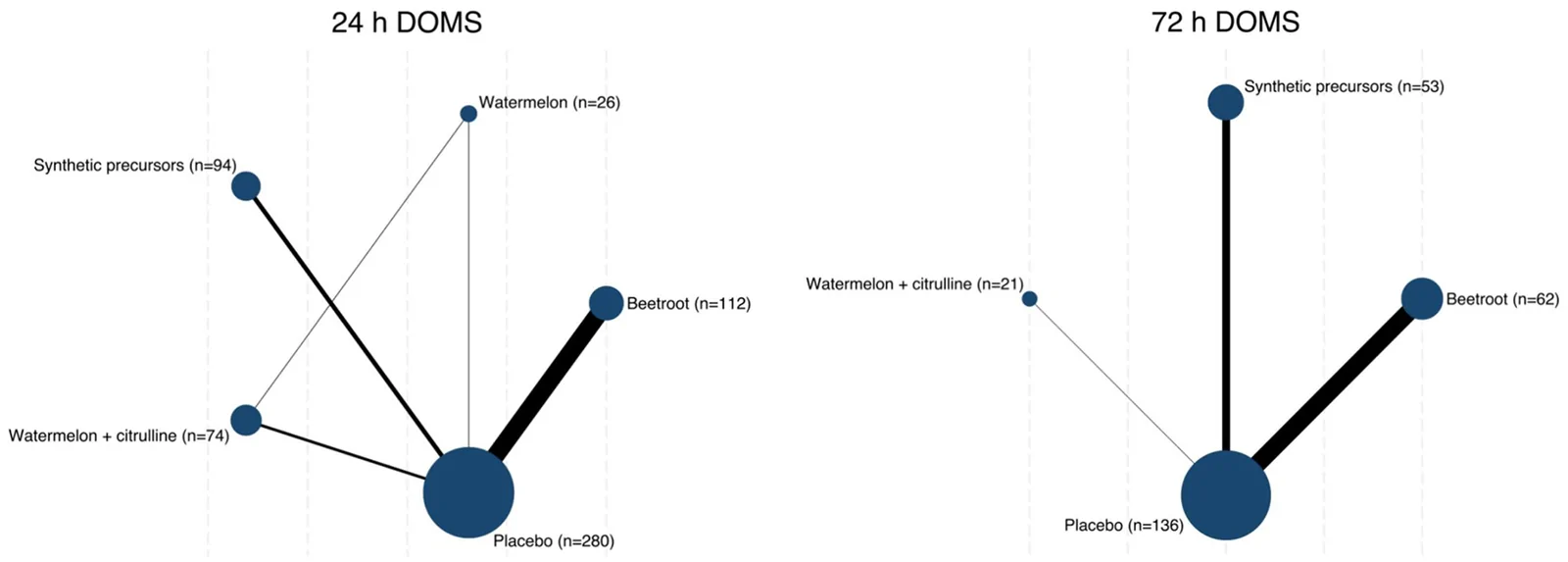
Network plots for 24-h and 72-h DOMS. Node labels: Beetroot, beetroot-derived nitrate; Watermelon, watermelon-derived citrulline; Synthetic precursors, synthetic or isolated amino acid precursors; Watermelon + citrulline, watermelon plus added citrulline. Placebo was the reference treatment.

**Figure 5:**
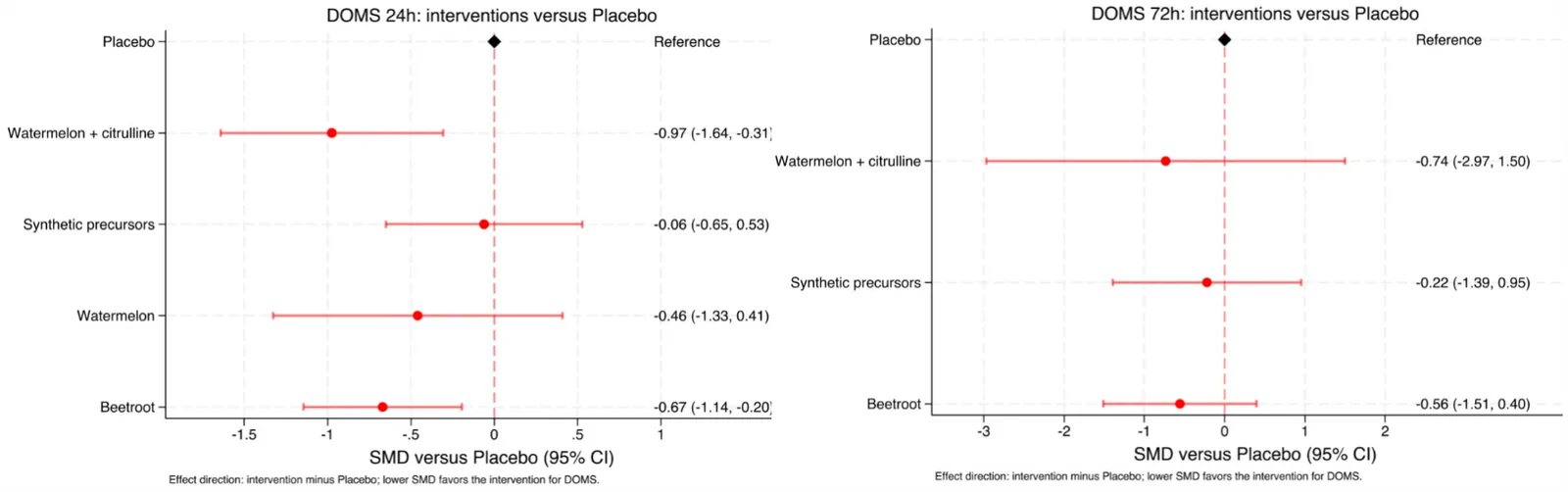
Forest plots of relative treatment effects for 24-h and 72-h DOMS vs. placebo. Node labels: Beetroot, beetroot-derived nitrate; Watermelon, watermelon-derived citrulline; Synthetic precursors, synthetic or isolated amino acid precursors; Watermelon + citrulline, watermelon plus added citrulline. Placebo was the reference treatment.

For the 72 h DOMS outcome, 11 studies involving 195 participants and four intervention nodes were included. The network again remained fully connected, with placebo as the common comparator and no isolated nodes (Figure 4). All active interventions showed a general tendency to reduce DOMS at 72 h compared with placebo, but none reached statistical significance (Figure 5B). According to the SUCRA rankings, beetroot-derived nitrate and watermelon plus added citrulline ranked highest, with SUCRA values of 66.0% and 64.6%, respectively. Synthetic or isolated amino acid precursors ranked next (SUCRA = 44.4%), whereas placebo ranked last (SUCRA = 25.0%) (Figure S5.3, Table S5.3 in Supplementary Appendix 5). The league table likewise showed no significant pairwise differences, indicating that the relative benefits of these interventions for reducing muscle soreness at 72 h remain uncertain (Table S6.3 in Supplementary Appendix 6).

### Subgroup analysis

#### Subgroup analysis of 48-h DOMS by training status

In the subgroup of trained participants and athletes, eight studies involving 147 participants and four treatment nodes were included. Beetroot-derived nitrate was the only intervention that showed a statistically significant benefit over placebo for 48 h DOMS (SMD = −1.92, 95% CI −3.33 to −0.51) (Figure S10.1 in Supplementary Appendix 10). Watermelon plus added citrulline and watermelon-derived citrulline also showed favorable trends, but neither comparison reached statistical significance. The league table supported these findings, showing a significant benefit for beetroot-derived nitrate over placebo, with no significant differences observed among the active interventions (Table S10.1 in Supplementary Appendix 10). In the subgroup of untrained and recreationally active participants, 10 studies involving 182 participants and five treatment nodes were included. None of the active interventions differed significantly from placebo (Figure S10.2 in Supplementary Appendix 10). Beetroot-derived nitrate and synthetic or isolated amino acid precursors showed point estimates favoring reduced DOMS, whereas the effects of watermelon-derived citrulline and watermelon plus added citrulline were close to the null. Consistent with the forest plot, the league table revealed no significant pairwise differences between active interventions, suggesting that their relative effects on 48 h DOMS remain uncertain within this subgroup (Table S10.2 in Supplementary Appendix 10).

#### Subgroup analysis of 48-h DOMS by supplementation duration

In the acute supplementation subgroup, eight studies involving 150 participants and five treatment nodes were included. Beetroot-derived nitrate was the only intervention associated with a statistically significant reduction in 48 h DOMS compared with placebo (SMD = −2.75, 95% CI −5.35 to −0.14) (Figure S10.3 in Supplementary Appendix 10). The other interventions, including watermelon plus added citrulline, watermelon-derived citrulline, and synthetic or isolated amino acid precursors, showed point estimates favoring reduced DOMS, but none reached statistical significance. The league table supported these findings and was consistent with the forest plot (Table S10.3 in Supplementary Appendix 10). In the multi-day supplementation subgroup, 10 studies involving 179 participants and three treatment nodes were included. Beetroot-derived nitrate significantly reduced 48 h DOMS compared with placebo (SMD = −0.99, 95% CI −1.73 to −0.24), whereas synthetic or isolated amino acid precursors did not show a significant effect (Figure S10.4 in Supplementary Appendix 10). The league table was consistent with the forest plot, with the point estimate favoring beetroot-derived nitrate over synthetic or isolated amino acid precursors. However, the difference between the two active interventions was not statistically significant (Table S10.4 in Supplementary Appendix 10).

### Sensitivity analyses

Sensitivity analyses for 48-h DOMS were broadly consistent with the primary findings (Tables S9.1–S9.2 in Supplementary Appendix 9). Beetroot-derived nitrate generally remained associated with lower DOMS, although the 95% CIs crossed the null when real-world or sport-specific mixed models were excluded and when the analysis was restricted to laboratory-controlled lower-limb mechanical EIMD models. Estimates for watermelon-derived citrulline, synthetic or isolated amino acid precursors, and watermelon plus added citrulline changed little across the sensitivity analyses, with no clear evidence of an effect. Varying the assumed within-subject correlation in crossover trials (*r* = 0.3, 0.7, and 0.9) produced only small changes in the estimates and did not materially change the overall interpretation.

## Discussion

### Primary findings

This network meta-analysis suggests that the effects of nitric oxide precursor interventions on post-exercise DOMS may differ across recovery time points and intervention types. Statistically significant benefits vs. placebo were observed for watermelon plus added citrulline and beetroot-derived nitrate at 24 h, and for beetroot-derived nitrate at 48 h, whereas no active intervention showed a significant benefit at 72 h. Evidence for differences between active interventions was limited, with only one statistically significant network comparison at 24 h, favoring watermelon plus added citrulline over synthetic or isolated amino acid precursors. This finding should be interpreted cautiously given the limited direct head-to-head evidence. SUCRA rankings should therefore be interpreted as exploratory rather than as evidence of superiority.

Subgroup and sensitivity analyses generally supported the primary findings, although some variation was observed across participant groups and exercise models. Beetroot-derived nitrate showed a significant benefit vs. placebo in the trained subgroup but not in the untrained subgroup, and the association was no longer statistically significant when real-world or sport-specific mixed models were excluded or when analyses were restricted to laboratory-controlled lower-limb mechanical EIMD models. Overall, these subgroup and sensitivity findings should therefore be interpreted cautiously.

### Comparison with previous research

Overall, the present findings are broadly consistent with previous research on nitric oxide precursor supplementation and post-exercise DOMS. However, the network meta-analysis approach allows different intervention nodes to be evaluated within a common framework and provides additional information on differences in estimated effects across recovery time points, although comparisons between active interventions remain largely indirect. Previous reviews have suggested that beetroot and inorganic nitrate may benefit some outcomes related to exercise-induced muscle damage, but that their effects on DOMS are weak or inconsistent (57, 58). The present results generally support this view, while also indicating that part of the inconsistency may reflect inadequate consideration of recovery timing in earlier analyses. Many previous reviews combined results from 24, 48, and 72 h, which may have obscured differences across recovery time points (8). In contrast, our time-specific analyses showed that beetroot-derived nitrate was associated with a significant reduction in DOMS vs. placebo at 24 h and 48 h, but not at 72 h. This pattern suggests that pooling outcomes across different recovery time points may partly contribute to the inconsistency reported previously. Evidence for L-citrulline and watermelon is similarly inconsistent. Systematic reviews and meta-analyses of citrulline-based supplementation have reported heterogeneous or equivocal effects, including for endurance, aerobic, and strength-related performance outcomes (59, 60). Within the post-exercise recovery literature, some studies have suggested that citrulline-related effects may be more apparent during the early recovery period and diminish by 72 h (61). Our findings are broadly consistent with this pattern. Watermelon plus added citrulline was associated with a significant reduction in DOMS vs. placebo at 24 h, but this effect was not evident at later time points. These findings suggest that recovery timing and variation in intervention formulation should be considered when interpreting citrulline-related effects.

In the present analysis, beetroot-derived nitrate and watermelon plus added citrulline showed favorable estimates or rankings at specific recovery time points. However, evidence for differences between active interventions was limited, with only one statistically significant network comparison at 24 h, favoring watermelon plus added citrulline over synthetic or isolated amino acid precursors. Given the predominantly placebo-centered network and limited direct head-to-head evidence, this isolated finding should be interpreted cautiously and should not be considered evidence of overall superiority. Differences in formulation and food matrix may nevertheless be relevant, because food-based products can provide not only the predominant NO-related precursor but also polyphenols, vitamins, and other bioactive compounds (15). These constituents could plausibly contribute to the observed responses, particularly given previous evidence for the antioxidant and anti-inflammatory properties of watermelon and beetroot (18, 62, 63). However, the present analysis cannot disentangle the potential contribution of these components from differences in precursor type, dose, formulation, study population, and exercise model.

### Potential mechanisms underlying time-dependent effects

The variation in treatment estimates across recovery time points may partly reflect changes in the biological processes contributing to soreness during recovery from exercise-induced muscle damage. In the early phase of recovery, mechanical disruption and metabolic disturbance predominate, whereas later stages may involve a greater contribution from secondary processes such as inflammation, oxidative stress, and local oedema (64). Within this framework, the associations observed for watermelon plus added citrulline and beetroot-derived nitrate at 24 h, and for beetroot-derived nitrate at 48 h may be biologically plausible in view of differences in their predominant NO-related precursors and pathways (13, 65). At 24 h, citrulline may increase arginine availability and thereby support nitric oxide production through nitric oxide synthase-dependent pathways (66, 67). This may support local perfusion and metabolic recovery, while watermelon may provide additional bioactive compounds such as lycopene and vitamin C (5). These characteristics could plausibly contribute to the early recovery pattern observed with watermelon plus added citrulline. By 48 h, DOMS commonly reaches its peak and may coincide with greater inflammatory activity, oxidative stress, oedema, and changes in local oxygen availability (2, 22). Under hypoxic or acidic conditions, the nitrate–nitrite–nitric oxide pathway may provide an alternative route for nitric oxide generation that is less dependent on nitric oxide synthase activity (68). This pathway may therefore be relevant to the 48 h effect observed with beetroot-derived nitrate. In addition, beetroot contains betalains and polyphenols with potential antioxidant and anti-inflammatory properties (69), which may complement nitrate-related effects during recovery. Taken together, these mechanisms provide a plausible biological framework for interpreting the observed time-dependent patterns. However, their specific contributions to DOMS recovery cannot be established from the present clinical evidence, and further studies incorporating mechanistic biomarkers are needed to clarify the biological processes underlying these time-specific effects.

### Strengths, robustness, and methodological limitations

#### Strengths and robustness

This study has several methodological strengths. A key strength is the use of network meta-analysis to evaluate distinct nitric oxide precursor intervention nodes within a common framework while preserving the time-specific nature of DOMS. The review followed PRISMA 2020 and PRISMA-NMA guidance and was registered in PROSPERO. The search strategy was comprehensive and included major bibliographic databases, ACSM and ECSS conference abstracts, and ClinicalTrials.gov. Risk of bias was assessed using RoB 2, and the certainty of the network evidence was evaluated using CINeMA. The use of multivariate random-effects models allowed direct and indirect evidence to be synthesized, with consistency assessed using global tests and node-splitting where the network structure permitted, while SUCRA rankings were interpreted alongside effect estimates and treated as exploratory. Subgroup and sensitivity analyses further examined whether the findings varied according to training status, supplementation duration, EIMD induction models, outcome definitions, and alternative within-subject correlation assumptions for crossover trials. For crossover trials, estimates were broadly similar across alternative within-subject correlation assumptions, while some results varied under specific analytical restrictions, highlighting the remaining uncertainty in the evidence.

### Limitations

Several limitations should be considered when interpreting these findings. First, the evidence network was relatively sparse and relied mainly on placebo-controlled comparisons, with few direct comparisons between active interventions. As a result, many treatment comparisons were informed largely by indirect evidence and depended on the comparability of the included studies. Consistency was assessed using both overall and local approaches where possible, but the limited network structure, including the absence of a closed loop at 72 h, restricted these assessments. SUCRA rankings and differences between active interventions should therefore be interpreted cautiously rather than as evidence of superiority. Second, substantial heterogeneity was present across recovery time points, particularly at 48 and 72 h. This may reflect differences in participant training status, muscle-damaging exercise protocols, supplement type, dose, timing, duration, and DOMS assessment methods. Interventions also varied in formulation, food matrix, and other bioactive components. Although standardized mean differences allowed different DOMS measures to be combined, subjective soreness ratings and pressure pain thresholds capture related but distinct aspects of pain. Excluding pressure pain threshold outcomes produced a broadly similar pattern, but residual clinical and measurement heterogeneity is likely. Several crossover trials also lacked paired variance data and required an assumed within-subject correlation; estimates were broadly similar under alternative assumptions, although some uncertainty remains. Third, despite the broad search strategy, some less commonly described nitrate-rich foods, nitrate salts, or formulations may have been missed. In addition, CINeMA rated the certainty of evidence for several key comparisons as low, largely because of imprecision, heterogeneity, and inconsistency in parts of the network. These limitations reduce confidence in the precision of the estimates. The biological mechanisms proposed to explain patterns across recovery time points were also not directly tested and should therefore be regarded as plausible rather than established. Future adequately powered head-to-head randomized trials using more standardized exercise protocols, supplementation regimens, and DOMS measures are needed to strengthen the evidence base.

### Clinical implications and future research directions

The present findings suggest that the effects of nitric oxide precursor supplementation on post-exercise DOMS may differ across recovery time points. Recovery timing may therefore be relevant when interpreting the effects of these supplements, rather than assuming a uniform effect throughout recovery. Overall, lower DOMS vs. placebo was observed with watermelon plus added citrulline and beetroot-derived nitrate at 24 h, and with beetroot-derived nitrate at 48 h. These results highlight the importance of considering both recovery timing and supplement formulation when interpreting their effects.

Future research should focus on adequately powered head-to-head randomized controlled trials to directly compare different NO precursors and reduce the uncertainty associated with the current reliance on indirect evidence. Biomarker assessment at key recovery time points may help clarify the biological mechanisms underlying the patterns observed across recovery time points (70). In addition, because women remain underrepresented in the current evidence base, future studies should place greater emphasis on female athletes under well-controlled physiological conditions to improve the generalisability of the findings (71, 72).

## Conclusion

This network meta-analysis suggests that nitric oxide precursor supplementation may show different patterns across recovery time points, with evidence of lower DOMS for watermelon plus added citrulline and beetroot-derived nitrate at 24 h and for beetroot-derived nitrate at 48 h, but limited evidence at 72 h or between active interventions. Given the sparse and heterogeneous evidence base and low certainty of some comparisons, adequately powered head-to-head trials are needed to confirm these findings.

## Data Availability

The original contributions presented in the study are included in the article/Supplementary material, further inquiries can be directed to the corresponding author.
