# Supplementary material for: Nitric oxide precursor interventions for post-exercise delayed onset muscle soreness: a systematic review and network meta-analysis of randomized placebo-controlled trials
Source: Front Nutr. 2026 Oct 15;13:1868555. doi: 10.3389/fnut.2026.1868555 (PMC13718329; doi:10.3389/fnut.2026.1868555)
Supplement: Supplementary file 1 [file Data_Sheet_1.pdf]

Supplementary Appendix

Nitric Oxide Precursor Interventions for Post-Exercise Delayed Onset Muscle Soreness: A Systematic Review and Network Meta-Analysis of Randomised Placebo-Controlled Trials

Kai-Xuan Xu [1], Shuai Guo[1,*] , Zhen-Fan Xie[2], Yu-Wei Xiao[3]

Table of contents

Appendix 1: Search strategy 2

Appendix 2: Variables extracted from the included studies........ 6

Appendix 3: Risk of bias of randomized clinical trials........ 10

Appendix 4: Evaluation of inconsistency and heterogeneity 11

Appendix 5: SUCRA and cumulative probability plots........ 14

Appendix 6: Network meta-analysis league tables of summary estimates........ 17

Appendix 7: CINeMA Assessment 20

Appendix 8: Funnel plots........ 24

Appendix 9: Sensitivity analyses 26

Appendix 10: Subgroup analysis of training status........ 28

Appendix 11: PRISMA checklist........ 32

Appendix 1: Search strategy

Table S1. Search strategy of PubMed

#	Searches
**1**	(((("nitric oxide precursor*"[Title/Abstract]) OR ("nitric oxide donor*"[Title/Abstract])) OR ("dietary nitrate*"[Title/Abstract])) OR ("inorganic nitrate*"[Title/Abstract])) OR ("nitrate supplementation"[Title/Abstract]) OR ("nitric oxide bioavailability"[Title/Abstract]) OR ("nitric oxide pathway"[Title/Abstract])
**2**	(((((((((("beetroot"[Title/Abstract]) OR ("beet root"[Title/Abstract])) OR ("beet juice"[Title/Abstract])) OR ("beetroot juice"[Title/Abstract])) OR ("Beta vulgaris"[Title/Abstract])) OR ("red beetroot"[Title/Abstract])) OR ("watermelon"[Title/Abstract])) OR ("watermelon juice"[Title/Abstract])) OR ("Citrullus lanatus"[Title/Abstract])) OR ("watermelon extract"[Title/Abstract])) OR ("L-arginine"[Title/Abstract]) OR ("L arginine"[Title/Abstract])) OR (arginine[Title/Abstract]) OR ("L-citrulline"[Title/Abstract]) OR ("L citrulline"[Title/Abstract]) OR (citrulline[Title/Abstract]) OR ("citrulline malate"[Title/Abstract]) OR ("citrulline-malate"[Title/Abstract]) OR ("CM supplementation"[Title/Abstract])
**3**	(((("delayed onset muscle soreness"[Title/Abstract]) OR ("DOMS"[Title/Abstract])) OR ("muscle soreness"[Title/Abstract])) OR ("exercise-induced muscle damage"[Title/Abstract])) OR ("EIMD"[Title/Abstract]) OR ("muscle damage"[Title/Abstract]) OR ("muscle pain"[Title/Abstract]) OR ("delayed muscle soreness"[Title/Abstract]) OR ("skeletal muscle damage"[Title/Abstract]) OR ("muscle injury"[Title/Abstract]) OR ("myalgia"[MeSH Terms])
**4**	((("resistance exercise"[Title/Abstract]) OR ("eccentric exercise"[Title/Abstract])) OR ("eccentric contraction*"[Title/Abstract])) OR ("muscle-damaging exercise"[Title/Abstract]) OR ("downhill running"[Title/Abstract]) OR ("drop jump*"[Title/Abstract]) OR ("plyometric*"[Title/Abstract]) OR ("exercise-induced damage"[Title/Abstract]) OR ("acute exercise"[Title/Abstract]) OR ("exercise protocol"[Title/Abstract]) OR ("Exercise"[MeSH Terms])
**5**	#1 OR #2
**6**	#3 OR #4
**7**	#5 AND #6
**8**	((("meta-analysis as topic"[MeSH Terms]) OR ("meta-analysis"[Publication Type])) OR ("systematic review"[Publication Type])) OR ("meta-analysis"[Title/Abstract]) OR ("systematic review"[Title/Abstract])
**9**	#7 NOT #8
**10**	((((((((("randomized controlled trial"[Publication Type]) OR ("controlled clinical trial"[Publication Type])) OR ("clinical trials as topic"[MeSH Terms])) OR ("random allocation"[MeSH Terms])) OR ("randomized controlled trials as topic"[MeSH Terms])) OR ("clinical trial"[Publication Type])) OR ("clinical trial"[Text Word])) OR ("randomized"[Text Word])) OR ("randomly"[Text Word])) OR ("placebo"[Text Word]) OR ("double blind*"[Text Word]) OR ("single blind*"[Text Word]) OR ("crossover"[Text Word]) OR ("cross-over"[Text Word])
**11**	#9 AND #10

Table S2. Search strategy of Web of Science: Science Citation Index Expanded

#	Searches
**1**	TS=("nitric oxide precursor*" OR "nitric oxide donor*" OR "dietary nitrate*" OR "inorganic nitrate*" OR "nitrate supplementation" OR "nitric oxide bioavailability" OR "nitric oxide pathway")
**2**	TS=(beetroot OR "beet root" OR "beet juice" OR "beetroot juice" OR "Beta vulgaris" OR "red beetroot" OR watermelon OR "watermelon juice" OR "Citrullus lanatus" OR "watermelon extract" OR "L-arginine" OR "L arginine" OR arginine OR "L-citrulline" OR "L citrulline" OR citrulline OR "citrulline malate" OR "citrulline-malate" OR "CM supplementation")
**3**	TS=("delayed onset muscle soreness" OR DOMS OR "muscle soreness" OR "exercise-induced muscle damage" OR EIMD OR "muscle damage" OR "muscle pain" OR "delayed muscle soreness" OR "skeletal muscle damage" OR "muscle injury" OR myalgia)
**4**	TS=("resistance exercise" OR "eccentric exercise" OR "eccentric contraction*" OR "muscle-damaging exercise" OR "downhill running" OR "drop jump*" OR plyometric* OR "exercise -induced damage" OR "acute exercise" OR "exercise protocol")
**5**	#1 OR #2
**6**	#3 OR #4
**7**	#5 AND #6
**8**	TS=("meta-analysis" OR "systematic review") OR DT=("Meta-Analysis" OR "Systematic Review")
**9**	#7 NOT #8
**10**	TS=(randomized OR randomly OR placebo OR "double blind*" OR "single blind*" OR crossover OR "cross-over" OR "clinical trial*" OR "controlled trial*" OR "randomised" OR "randomly allocated") OR DT=("Randomized Controlled Trial" OR "Clinical Trial")
**11**	#9 AND #10

Table S3. Search strategy of Cochrane Central Register of Controlled Trials (CENTRAL)

#	Searches
**1**	(nitric NEXT oxide NEXT precursor*):ti,ab,kw OR (nitric NEXT oxide NEXT donor*):ti,ab,kw OR (dietary NEXT nitrate*):ti,ab,kw OR (inorganic NEXT nitrate*):ti,ab,kw OR ("nitrate supplementation"):ti,ab,kw OR ("nitric oxide bioavailability"):ti,ab,kw OR ("nitric oxide pathway"):ti,ab,kw
**2**	(beetroot):ti,ab,kw OR ("beet root"):ti,ab,kw OR ("beet juice"):ti,ab,kw OR ("beetroot juice"):ti,ab,kw OR ("Beta vulgaris"):ti,ab,kw OR ("red beetroot"):ti,ab,kw OR (watermelon):ti,ab,kw OR ("watermelon juice"):ti,ab,kw OR ("Citrullus lanatus"):ti,ab,kw OR ("watermelon extract"):ti,ab,kw OR ("L-arginine"):ti,ab,kw OR ("L arginine"):ti,ab,kw OR (arginine):ti,ab,kw OR ("L-citrulline"):ti,ab,kw OR ("L citrulline"):ti,ab,kw OR (citrulline):ti,ab,kw OR ("citrulline malate"):ti,ab,kw OR ("citrulline-malate"):ti,ab,kw OR ("CM supplementation"):ti,ab,kw
**3**	("delayed onset muscle soreness"):ti,ab,kw OR (DOMS):ti,ab,kw OR ("muscle soreness"):ti,ab,kw OR ("exercise-induced muscle damage"):ti,ab,kw OR (EIMD):ti,ab,kw OR ("muscle damage"):ti,ab,kw OR ("muscle pain"):ti,ab,kw OR ("delayed muscle soreness"):ti,ab,kw OR ("skeletal muscle damage"):ti,ab,kw OR ("muscle injury"):ti,ab,kw OR [mh Myalgia]
**4**	("resistance exercise"):ti,ab,kw OR ("eccentric exercise"):ti,ab,kw OR (eccentric NEXT contraction*):ti,ab,kw OR ("muscle-damaging exercise"):ti,ab,kw OR ("downhill running"):ti,ab,kw OR (drop NEXT jump*):ti,ab,kw OR (plyometric*):ti,ab,kw OR ("exercise-induced damage"):ti,ab,kw OR ("acute exercise"):ti,ab,kw OR ("exercise protocol"):ti,ab,kw OR [mh Exercise]
**5**	#1 OR #2
**6**	#3 OR #4
**7**	#5 AND #6

Table S4. Search strategy of Embase

#	Searches
**1**	'nitric oxide precursor*':ti,ab OR 'nitric oxide donor*':ti,ab OR 'dietary nitrate*':ti,ab OR 'inorganic nitrate*':ti,ab OR 'nitrate supplementation':ti,ab OR 'nitric oxide bioavailability':ti,ab OR 'nitric oxide pathway':ti,ab
**2**	beetroot:ti,ab OR 'beet root':ti,ab OR 'beet juice':ti,ab OR 'beetroot juice':ti,ab OR 'Beta vulgaris':ti,ab OR 'red beetroot':ti,ab OR watermelon:ti,ab OR 'watermelon juice':ti,ab OR 'Citrullus lanatus':ti,ab OR 'watermelon extract':ti,ab OR 'L-arginine':ti,ab OR 'L arginine':ti,ab OR arginine:ti,ab OR 'L-citrulline':ti,ab OR 'L citrulline':ti,ab OR citrulline:ti,ab OR 'citrulline malate':ti,ab OR 'citrulline-malate':ti,ab OR 'CM supplementation':ti,ab
**3**	'delayed onset muscle soreness':ti,ab OR DOMS:ti,ab OR 'muscle soreness':ti,ab OR 'exercise induced muscle damage':ti,ab OR EIMD:ti,ab OR 'muscle damage':ti,ab OR 'muscle pain':ti,ab OR 'delayed muscle soreness':ti,ab OR 'skeletal muscle damage':ti,ab OR 'muscle injury':ti,ab OR 'myalgia'/exp
**4**	'resistance exercise':ti,ab OR 'eccentric exercise':ti,ab OR 'eccentric contraction*':ti,ab OR 'muscle damaging exercise':ti,ab OR 'downhill running':ti,ab OR 'drop jump*':ti,ab OR plyometric*:ti,ab OR 'exercise induced damage':ti,ab OR 'acute exercise':ti,ab OR 'exercise protocol':ti,ab OR 'exercise'/exp
**5**	#1 OR #2
**6**	#3 OR #4
**7**	#5 AND #6
**8**	'meta-analysis':ti,ab OR 'systematic review':ti,ab OR 'meta analysis'/exp OR 'systematic review'/exp
**9**	#7 NOT #8
**10**	'randomized controlled trial':ti,ab OR 'controlled clinical trial':ti,ab OR random*:ti,ab OR placebo:ti,ab OR 'double blind*':ti,ab OR 'single blind*':ti,ab OR crossover:ti,ab OR 'cross-over':ti,ab OR 'clinical trial*':ti,ab OR 'randomized controlled trial'/exp OR 'clinical trial'/exp
**11**	#9 AND #10

Appendix 2: Variables extracted from the included studies

Table S2.1: Variables extracted from the included studies

Data category	List of variables
Study	First author, publication year, country or region, study design, total sample size, and washout period for crossover studies
Participant characteristics	Sex, age, training status, competitive or athletic level, and other baseline participant characteristics when available
EIMD protocol	Predefined EIMD model category, exercise modality, target muscle region, exercise load or intensity, and session duration or volume
Intervention and comparator characteristics	intervention group classification, assigned NMA node, source or matrix of the NO-related precursor, key bioactive ingredient(s), formulation, per-serving dose, active ingredient content, daily frequency, supplementation timing, total intervention duration, and placebo/control description
Outcomes	DOMS assessment method, measurement scale or instrument, outcome direction, sampling time points, analysed sample size in each arm, and extractable summary statistics for each group, including mean, standard deviation, or data convertible to these values
Additional methodological information	Randomisation procedures, blinding, attrition or missing data, and additional study features relevant to risk-of-bias assessment, sensitivity analysis, or intervention classification

Table S2.2: Intervention, EIMD, and DOMS assessment characteristics of the included studies

Study ID	Intervention	Intervention node	Dose and formulation	Timing	Duration	Washout period	Comparator	EIMD model	EIMD protocol	DOMS assessment
2020 Daab	Beetroot powder dissolved in juice	Beetroot-derived nitrate	Juice; 150 mL; 250 mg nitrate	Twice daily for 7 days: 3 days before, exercise day, and 3 days after LIST	7 days	Yes; duration not reported	Matched placebo juice	LIST soccer-simulation model	Loughborough Intermittent Shuttle Test with walking, sprinting, and running at 55% and 95% VO2max; Intermittent sport-simulation intensity; ~90 min	NRS (0-10)
2024 Ahmadpour	Beetroot juice	Beetroot-derived nitrate	Juice; 220 mL; ~8.9 mmol nitrate/220 mL	2.5 h before EIMD; no post-exercise supplementation	1 day	7 days	Placebo juice	Sport-specific alpine skiing model	90-s box jumps, hexagon jumps, wall sit, three free-ski runs, dynamic warm-up, and two full slalom runs; High altitude, high-intensity slalom runs; 3 free-ski runs + 2 full slalom runs	VAS
2016 Tom Clifford	Beetroot juice	Beetroot-derived nitrate	Concentrated juice; H-BT 250 mL, ~250 mg nitrate, 194 mg betanin, 401.7 mg GAE	Started 30 min post-exercise; additional doses at 2 h and dinner on day 1; twice daily on days 2-3	3 days	NA	Matched placebo juice	Drop-jump model	0.6-m drop jumps with knee flexion to ~90 degrees followed by maximal vertical jump; Maximal-effort jumping; 5 x 20 repetitions; 100 jumps	PPT
2016 Tom Clifford	Beetroot juice	Beetroot-derived nitrate	Concentrated juice; 250 mL; >=143 mg nitrate; TEAC ~11.4 mmol/L	Started 30 min post-exercise; continued twice daily until the second sprint bout at 72 h	4 days	NA	Matched placebo juice	Repeated sprint/deceleration model	20 x 30-m maximal sprints with forced deceleration; Maximal effort; 20 sprints	PPT
2016 Tom Clifford	Beetroot beverage	Beetroot-derived nitrate	Juice/beverage; 250 mL; Not clearly stated in original Table 1; source file records dose/frequency but not nitrate content	Immediate post-marathon, 3 h post, and evening on day 1; three doses on day 2; morning dose on day 3	3 days	NA	Matched placebo beverage	Marathon model	Full marathon over mixed terrain including soft sand; Maximal race completion effort; One full marathon	VAS (0-100)
2017 Tom Clifford	Beetroot juice	Beetroot-derived nitrate	Juice; BTJ 250 mL; BTJ ~210 mg nitrate;	Started 30 min post-exercise; additional day-1 and recovery doses through 48 h	3 days	NA	Matched placebo juice	Drop-jump model	0.6-m drop jumps with knee flexion to ~90 degrees and maximal vertical jump; Maximal -effort jumping; 100 jumps	PPT

2023 Mohammad Hemmatinafar		Beetroot juice	Beetroot-derived nitrate	Juice; 50 mL per dose; ~0.205 mmol nitrate/dose	Started 2 h after EIMD; doses at 2, 6, 10, 14, 26, 30, 34, and 38 h	2 days	30 days	Matched placebo drink	Weighted vertical-jump model	Vertical jumps while wearing a vest equal to 10% body mass; Maximum jumps; 10 x 20 repetitions; 200 jumps	VAS (0-100)
2025 Alireza Daneshparvar		Beetroot juice/beverage	Beetroot-derived nitrate	Juice/beverage; 70 mL; 400 mg nitrate/70 mL	Single dose 2.5 h before trekking; no post-exercise supplementation	1 day	NA	Matched placebo beverage; water control	High-altitude trekking/downhill walking model	20-km round trip to 3720 m with 870-m elevation gain; High-altitude trekking; ~5 h ascent + ~4.5 h descent	VAS (0-100)
2013 Tarazon a-Díaz	(a)	watermelon juice	Watermelon-derived citrulline	Juice; 500 mL; 1.17 g L -citrulline/500 mL	Single dose 1 h before exercise	1 day	5 day	Matched fruit -flavored placebo juice	High-intensity cycle sprint model	Cycle ergometer repeated sprint test with 8 x 30-s maximal bouts; 4.5-5 kg resistance; maximal bouts; 11-min protocol plus warm-up	Likert scale (1-5)
	(b)	Watermelon plus added L-citrulline	Watermelon plus added citrulline	Enriched juice; 500 mL; 6.0 g L-citrulline/500 mL	Single dose 1 h before exercise	1 day	5 day	Matched placebo juice	High-intensity cycle sprint model	Cycle ergometer repeated sprint test with 8 x 30-s maximal bouts; 4.5-5 kg resistance; maximal bouts; 11-min protocol plus warm-up	Likert scale (1-5)
2017 Martínez-Sánchez		Watermelon plus added L-citrulline	Watermelon plus added citrulline	Juice; 500 mL; 3.45 g L -citrulline	Single dose 2 h before the half-marathon	1 day	14 day	Matched placebo	Half-marathon model	Complete half-marathon including uphill and downhill sections; Near anaerobic threshold; 21.097 km	Likert scale (1-5)
2017 Martín ez-Sánchez	(a)	watermelon juice	Watermelon-derived citrulline	Natural juice; 200 mL; 0.5 g L-citrulline	Single dose 1 h before half-squat exercise	1 day	7 day	Matched placebo beverage	Half-squat resistance model	8 x 8 half-squat at 8RM with 3-s eccentric phase and maximal concentric phase; 8RM; 64 total repetitions	Likert scale (1-5)
	(b)	Watermelon plus added L-citrulline	Watermelon plus added citrulline	Enriched juice; 200 mL; 3.3 g L-citrulline	Single dose 1 h before half-squat exercise	1 day	7 day	Matched placebo juice	Half-squat resistance model	8 x 8 half-squat at 8RM with 3-s eccentric phase and maximal concentric phase; 8RM; 64 total repetitions	Likert scale (1-5)

2010 Pérez-Guisado	Citrulline malate beverage	Synthetic/isolated amino-acid precursor	Beverage; 200 mL beverage; 8 g citrulline malate	Single dose 1 h before resistance exercise	1 day	7 day	Matched placebo juice	Chest resistance-training model	Bench press, incline bench press, incline flyes, and bench press sets to failure; 80% 1RM for presses; 60% 1RM for flyes; 16 sets to failure	Likert scale (1-5)
2025 Salem	Beetroot juice	Beetroot-derived nitrate	Powder dissolved in juice; 15 g on exercise/testing days; 5 g recovery doses; 15 g powder contains 450 mg nitrate	15 g 2 h before exercise; recovery doses at 24/48 h; 15 g again before 72-h testing	4 days	7 day	Matched placebo juice	Bench press and back squat resistance model	Bench press and back squat performed to failure at 60%, 70%, and 80% 1RM; Progressive 1RM loading; 18 sets to failure	NRS (0-10)
2017 Clifford	Beetroot concentrate	Beetroot-derived nitrate	Concentrated juice; H-BT 250 mL; ~250 mg nitrate	Immediately post-exercise, 2 h post, evening; then after 24-h and 48-h testing	3 days	NA	Cornstarch placebo	Drop-jump model	0.6-m drop jumps with knee flexion to 90 degrees followed by maximal vertical jump; Maximal-effort jumping; 5 x 20 repetitions; 100 jumps	PPT
2020 Blohm	Fortified watermelon beverage	Watermelon plus added citrulline	Bottled beverage; 355 mL; 780 mg L-citrulline	Single dose 45 min before submaximal cycling	1 day	⩾2 days	Citric acid placebo	Submaximal cycling model	Submaximal cycling to 80% predicted HRmax; Submaximal aerobic test; ~6.4-9.3 min	Likert scale (1-5)
2018 Andrade	L-arginine powder	Synthetic/isolated amino-acid precursor	Powder in water; 6 g; 6 g L-arginine	60 min before exercise; repeated 60 min before 24-, 48-, and 72-h testing	4 days	NA	Citric acid placebo	Lower-limb resistance model	Leg press and hack squat; 70% 1RM; 2 exercises x 3 sets x 8-12 reps	10-cm VAS
2018 Chappell	Citrulline malate powder	Synthetic/isolated amino-acid precursor	Powder in water/juice syrup; 8 g; 4.2 g citrulline + 3.8 g malate	Single dose 1 h before barbell-curl exercise	1 day	7 day	Cornstarch placebo	German volume training model	Barbell curls; 80% 1RM; 10 x 10 repetitions	100-mm VAS
2018 Chappell	Citrulline malate powder	Synthetic/isolated amino-acid precursor	Powder in water/juice syrup; 8 g; 4.21 g citrulline + 3.79 g malate	Single dose 1 h before knee-extension exercise	1 day	7 day	Placebo juice	Single-leg knee-extension model	Isokinetic single-leg knee extension; 70% concentric peak force; 10 x 10 repetitions	100-mm VAS
2017 da Silva	Citrulline malate powder	Synthetic/isolated amino-acid precursor	Powder in water; 6 g; 6 g citrulline malate	60 min before exercise and before 24-, 48-, and 72-h follow-up testing	4 days	7 day	Matched placebo juice	Lower-limb resistance model	Leg press and hack squat; 90% 10RM; 2 exercises x 3 sets x 8-12 reps	10-cm VAS

Appendix 3: Risk of bias of randomized clinical trials

Table S3: Risk of bias at the study level was assessed for all included randomized controlled trials using the Cochrane Risk of Bias 2 tool.

Unique ID	Randomization process	Deviations from intended interventions	Missing outcome data	Measurement of the outcome	Selection of the reported result	Over all
2025Salem	Low	Low	Low	Low	Low	Low
2024Ahmadpour	Low	Low	Low	Low	Low	Low
2016Tom Clifford	Low	Low	Low	Low	Low	Low
2016Tom Clifford	Low	Low	Low	Low	Low	Low
2016Tom Clifford	Low	Low	Low	Low	Low	Low
2017 Tom Clifford	Low	Low	Low	Low	Low	Low
2023Mohammad Hemmatinafar	Low	Low	Low	Low	Low	Low
2025Alireza Daneshparvar	Low	Low	Low	Low	Low	Low
2013Tarazona-Díaz	Low	Some concerns	Low	Low	Low	Some concerns
2017Martínez-Sánchez	Low	Low	Low	Low	Low	Low
2017Martínez-Sánchez	Low	Low	Low	Low	Low	Low
2010Pérez-Guisado	Low	Some concerns	Low	Low	Low	Some concerns
2020Daab	Some concerns	Some concerns	Low	Low	Low	High
2017Clifford	Low	Low	Low	Low	Low	Low
2020Blohm	Low	Low	Low	Low	Low	Low
2018Andrade	Low	Low	Low	Low	Low	Low
2018Chappell	Low	Low	Low	Low	Low	Low
2018Chappell	Low	Low	Low	Low	Low	Low
2017da Silva	Low	Low	Low	Low	Low	Low

Appendix 4: Evaluation of inconsistency and heterogeneity

Table S4.1: Table S4.1. Global inconsistency and between-study heterogeneity for DOMS at 24, 48, and 72 h

Outcome	Timepoint	Chi square	df	p	Tau^2
DOMS	24 h	0.18	1	0.6687	0.3585
DOMS	48 h	0.03	1	0.8568	1.0471
DOMS	72 h	0.42	1	0.5191	1.2337

Note: Chi-square, df, and p values were obtained from the global inconsistency test, whereas Tau^2 represents the estimated between-study heterogeneity. Local inconsistency was additionally explored using node-splitting at 24 h and 48 h, where closed loops provided both direct and indirect evidence for relevant comparisons. At 72 h, no closed loop was available to support a meaningful local inconsistency assessment. Because the networks were predominantly placebo-centred and the amount of independent closed-loop evidence was limited, the node-splitting results should be interpreted cautiously.

Table S4.2: Node-splitting assessment of local inconsistency for DOMS at 24 h

Comparison	Direct		Indirect		Difference		
	Coef.	Std. Err.	Coef.	Std. Err.	Coef.	Std. Err.	p>|z|
Beetroot vs. Placebo	0.670	0.242	0.339	105.163	0.330	105.163	0.997
Watermelon vs. Watermelon + citrulline	-0.598	0.500	0.011	1.310	-0.609	1.424	0.669
Watermelon vs. Placebo	0.546	0.501	-0.063	1.309	0.609	1.424	0.669
Synthetic precursors vs. Placebo	0.061	0.301	1.278	286.824	-1.217	286.824	0.997
Watermelon + citrulline vs. Placebo	0.974	0.341	0.937	316.570	0.037	316.570	1.000

Note: The p value tests the difference between direct and indirect estimates.

Table S4.3: Node-splitting assessment of local inconsistency for DOMS at 48 h

Comparison	Direct		Indirect		Difference		
	Coef.	Std. Err.	Coef.	Std. Err.	Coef.	Std. Err.	P>|z|
Beetroot vs. Placebo	1.180	0.372	0.142	111.731	1.038	111.732	0.993
Watermelon vs. Watermelon + citrulline	-0.458	0.813	0.037	2.595	-0.495	2.745	0.857
Watermelon vs. Placebo	0.133	0.773	-0.362	2.632	0.495	2.745	0.857
Synthetic precursors vs. Placebo	0.105	0.482	2.255	282.802	-2.150	282.802	0.994
Watermelon + citrulline vs. Placebo	0.505	0.624	2.456	365.320	-1.951	365.320	0.996

Note: The p value tests the difference between direct and indirect estimates.

Appendix 5: SUCRA and cumulative probability plots

Figure S5.1: Cumulative ranking curves for interventions on 48-H DOMS. A greater area under the curve indicates a higher likelihood of a more favorable ranking, corresponding to lower DOMS values at 48 h.

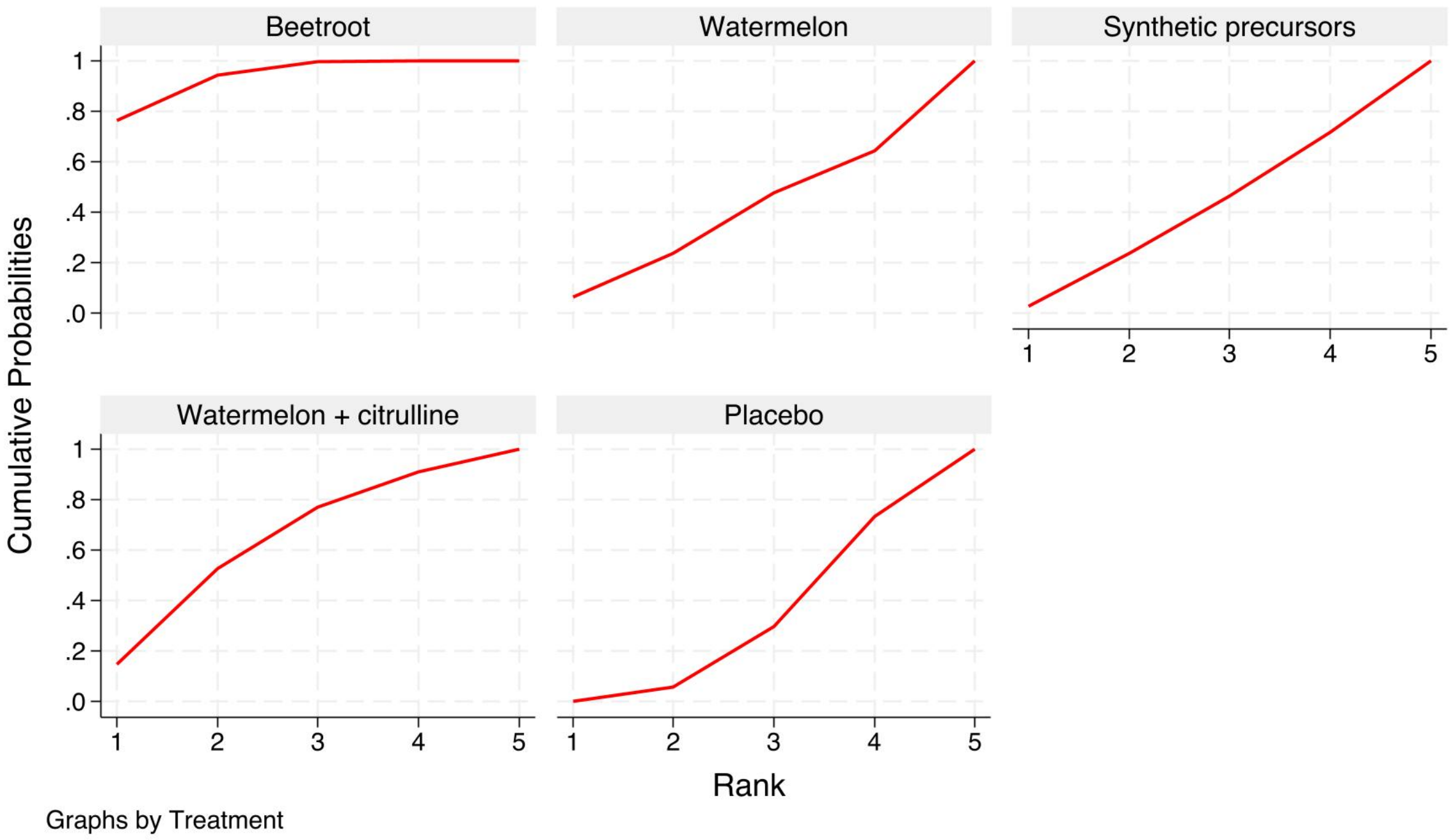

Table S5.1: SUCRA of the effects of the included interventions on 48-H DOMS.

Treatment	SUCRA	PrBest	MeanRank
Beetroot	92.5	76.4	1.30
Watermelon + citrulline	58.8	14.7	2.65
Synthetic precursors	36.1	2.6	3.56
Watermelon	35.5	6.3	3.58
Placebo	27.1	0.0	3.91

Abbreviations: SUCRA, surface under the cumulative ranking curve

Figure S5.2: Cumulative ranking curves for interventions on 24-H DOMS. A greater area under the curve indicates a higher likelihood of a more favorable ranking, corresponding to lower DOMS values at 24 h.

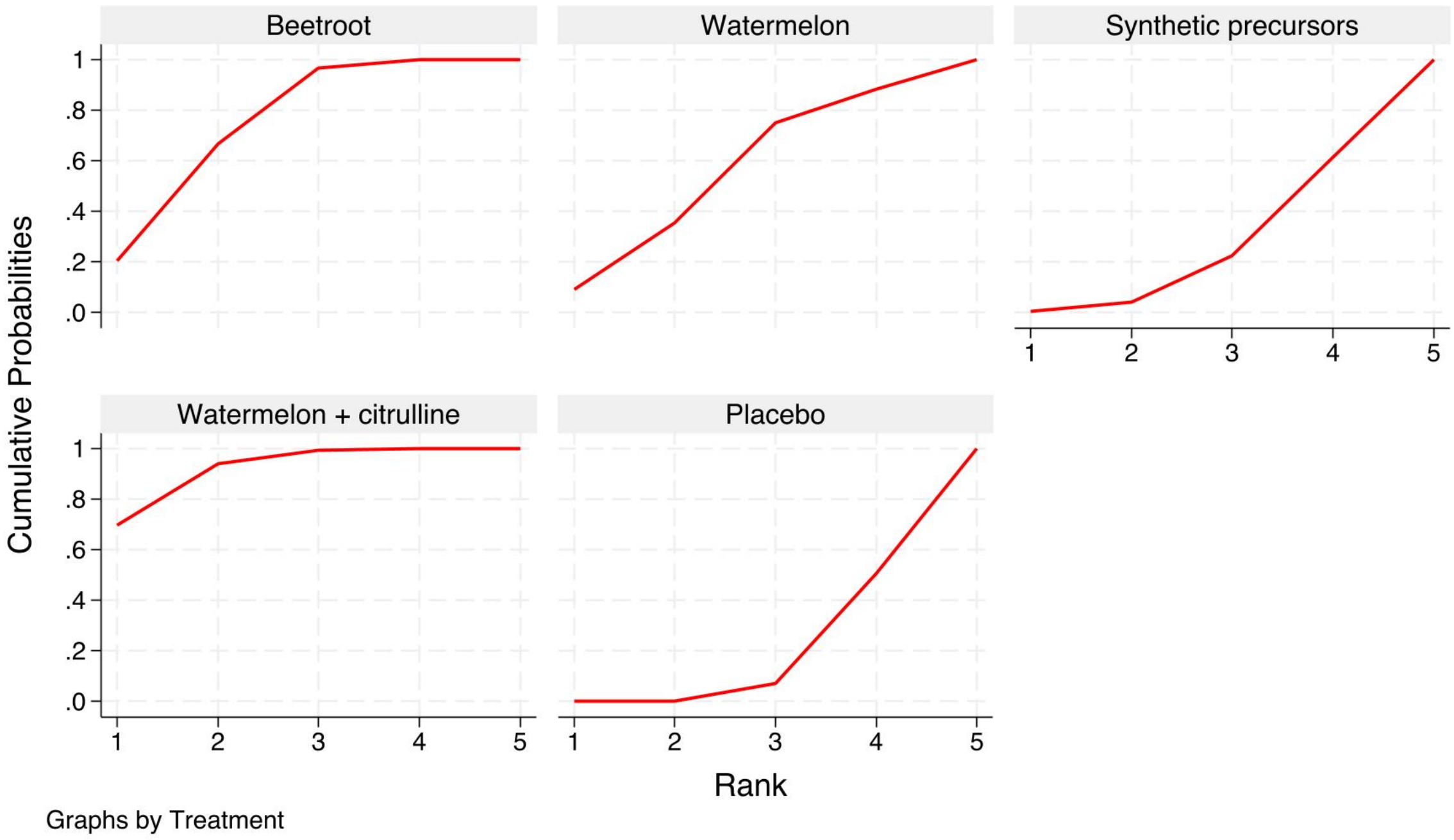

Table S5.2: SUCRA of the effects of the included interventions on 24-H DOMS.

Treatment	SUCRA	PrBest	MeanRank
Watermelon + citrulline	90.7	69.8	1.37
Beetroot	70.9	20.5	2.16
Watermelon	51.9	9.1	2.92
Synthetic precursors	22.1	0.6	4.12
Placebo	14.4	0.0	4.42

Abbreviations: SUCRA, surface under the cumulative ranking curve

Figure S5.3: Cumulative ranking curves for interventions on 72-H DOMS. A greater area under the curve indicates a higher likelihood of a more favorable ranking, corresponding to lower DOMS values at 72 h.

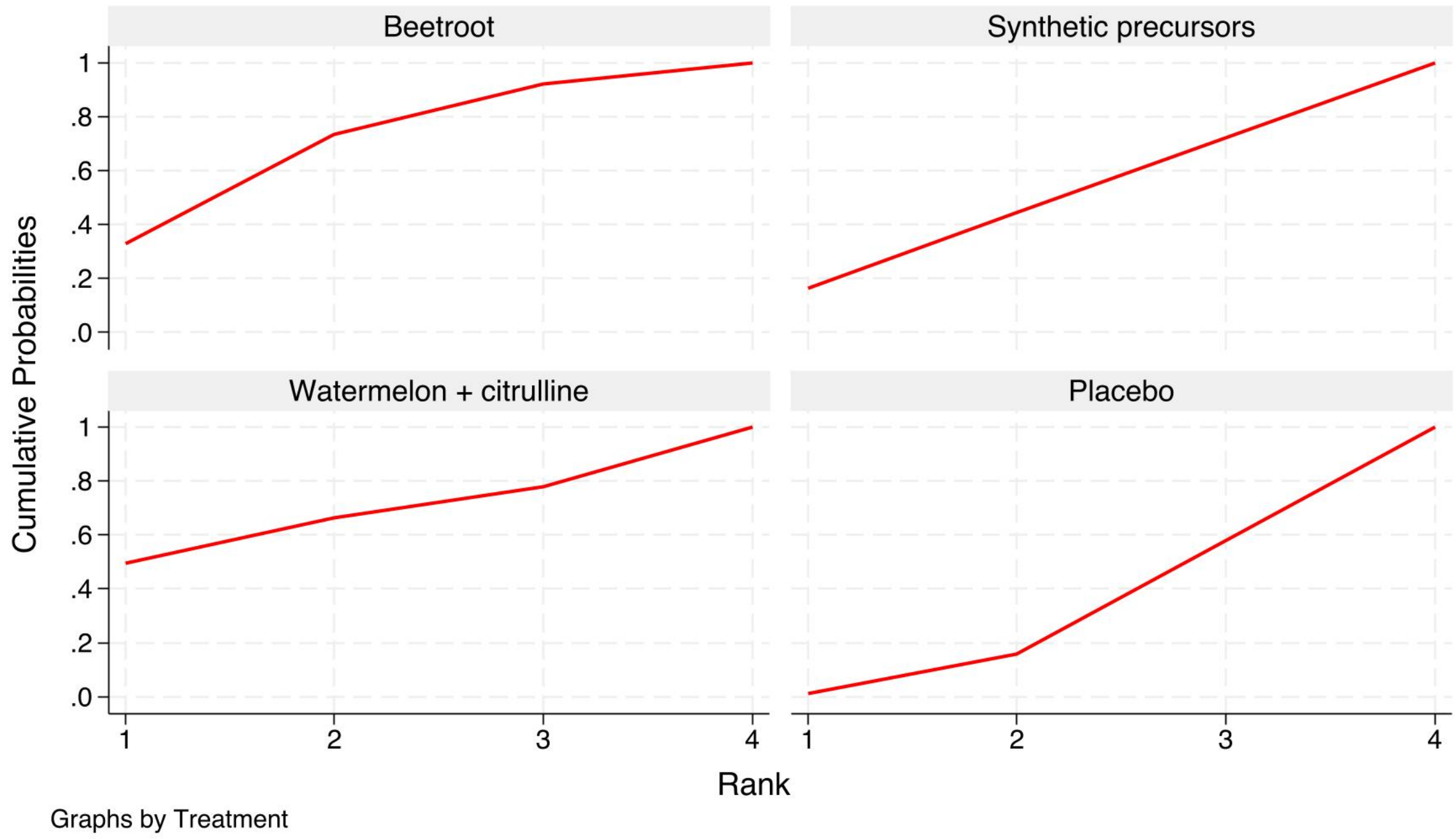

Table S5.3: SUCRA of the effects of the included interventions on 72-H delayed onset muscle soreness (DOMS).

Treatment	SUCRA	PrBest	MeanRank
Beetroot	66.0	32.8	2.02
Watermelon + citrulline	64.6	49.5	2.06
Synthetic precursors	44.4	16.5	2.67
Placebo	25.0	1.1	3.25

Abbreviations: SUCRA, surface under the cumulative ranking curve

Appendix 6. Network meta-analysis league tables of summary estimates

Table S6.1: DOMS at 48 h Post-Exercise

Each cell shows the standardized mean difference (SMD) with its 95% confidence interval (CI) for the column intervention compared with the row intervention. For DOMS, lower values indicate less pain; thus, an SMD < 0 favors the column intervention, whereas an SMD > 0 favors the row intervention. Statistically significant comparisons (95% CI not crossing zero) are color-coded: pink indicates favoring the column intervention, green indicates favoring the row intervention, and white indicates no significant difference.

Beetroot				
-1.09 (-2.64, 0.47)	Watermelon			
-1.08 (-2.27, 0.12)	0.01 (-1.66, 1.68)	Synthetic precursors		
-0.67 (-2.10, 0.75)	0.41 (-1.02, 1.84)	0.40 (-1.14, 1.95)	Watermelon + citrulline	
-1.18 (-1.91, -0.45)	-0.09 (-1.47, 1.28)	-0.11 (-1.05, 0.84)	-0.51 (-1.73, 0.72)	Placebo

Note: Only pink-shaded off-diagonal cells appear because all statistically significant comparisons favored the column intervention.

Table S6.2: DOMS at 24 h Post-Exercise

Each cell shows the standardized mean difference (SMD) with its 95% confidence interval (CI) for the column intervention compared with the row intervention. For DOMS, lower values indicate less pain; thus, an SMD < 0 favors the column intervention, whereas an SMD > 0 favors the row intervention. Statistically significant comparisons (95% CI not crossing zero) are color-coded: pink indicates favoring the column intervention, green indicates favoring the row intervention, and white indicates no significant difference.

**Beetroot**				
-0.21 (-1.20, 0.78)	**Watermelon**			
-0.61 (-1.36, 0.15)	-0.40 (-1.45, 0.65)	**Synthetic precursors**		
0.30 (-0.51, 1.12)	0.51 (-0.35, 1.38)	0.91 (0.02, 1.80)	**Watermelon + citrulline**	
-0.67 (-1.14, -0.20)	-0.46 (-1.33, 0.41)	-0.06 (-0.65, 0.53)	-0.97 (-1.64, -0.31)	**Placebo**

Table S6.3: DOMS at 72 h Post-Exercise

Each cell presents the standardized mean difference (SMD) and 95% confidence interval (95% CI) for the column intervention versus the row intervention. For DOMS, lower values indicate less pain; therefore, SMD < 0 favors the column intervention, whereas SMD > 0 favors the row intervention. Cells with statistically significant results (95% CI excluding zero) are colour-coded: pink shading indicates that the column intervention is favored (SMD < 0), green shading indicates that the row intervention is favored (SMD > 0), and white cells indicate no statistically significant difference.

**Beetroot**			
-0.34 (-1.85, 1.18)	**Synthetic precursors**		
0.18 (-2.25, 2.61)	0.51 (-2.01, 3.04)	**Watermelon + citrulline**	
-0.56 (-1.51, 0.40)	-0.22 (-1.39, 0.95)	-0.74 (-2.97, 1.50)	**Placebo**

Appendix 7: CINeMA Assessment

Evidence certainty for each network estimate was evaluated with CINeMA. Within-study bias was categorized as low, moderate, or high risk. Studies were rated as moderate risk when no domain was judged high risk, and as high risk when one or more domains were rated high risk. Detailed risk-of-bias assessments are shown in Appendix 3, and bias contributions for each pairwise comparison are presented in Figures S7.1 – S7.2.

Figure S7.1: Risk of bias contribution by intervention group for 48-H DOMS

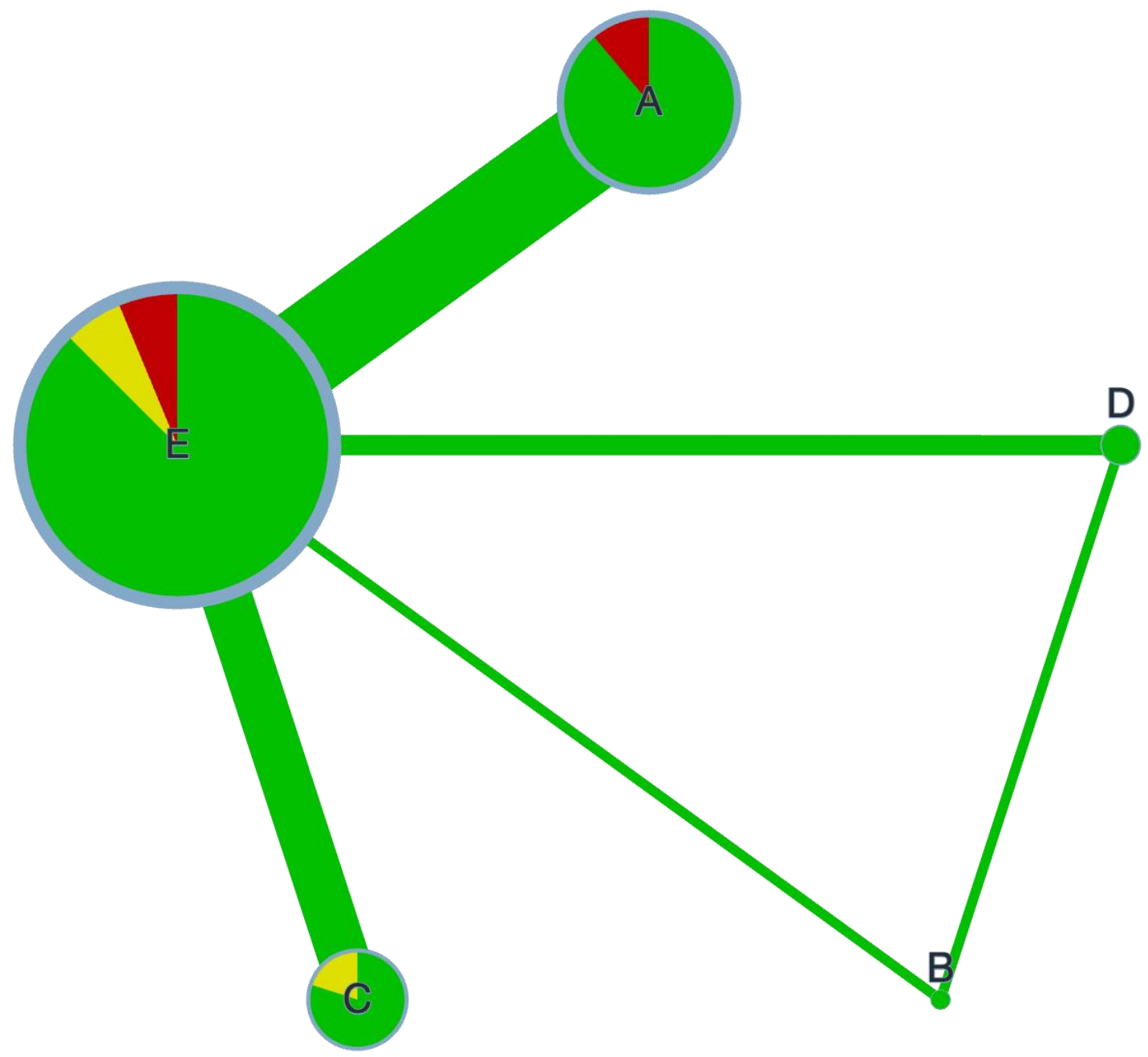

Figure S7.2: Overall risk of bias by treatment comparison in 48-h DOMS

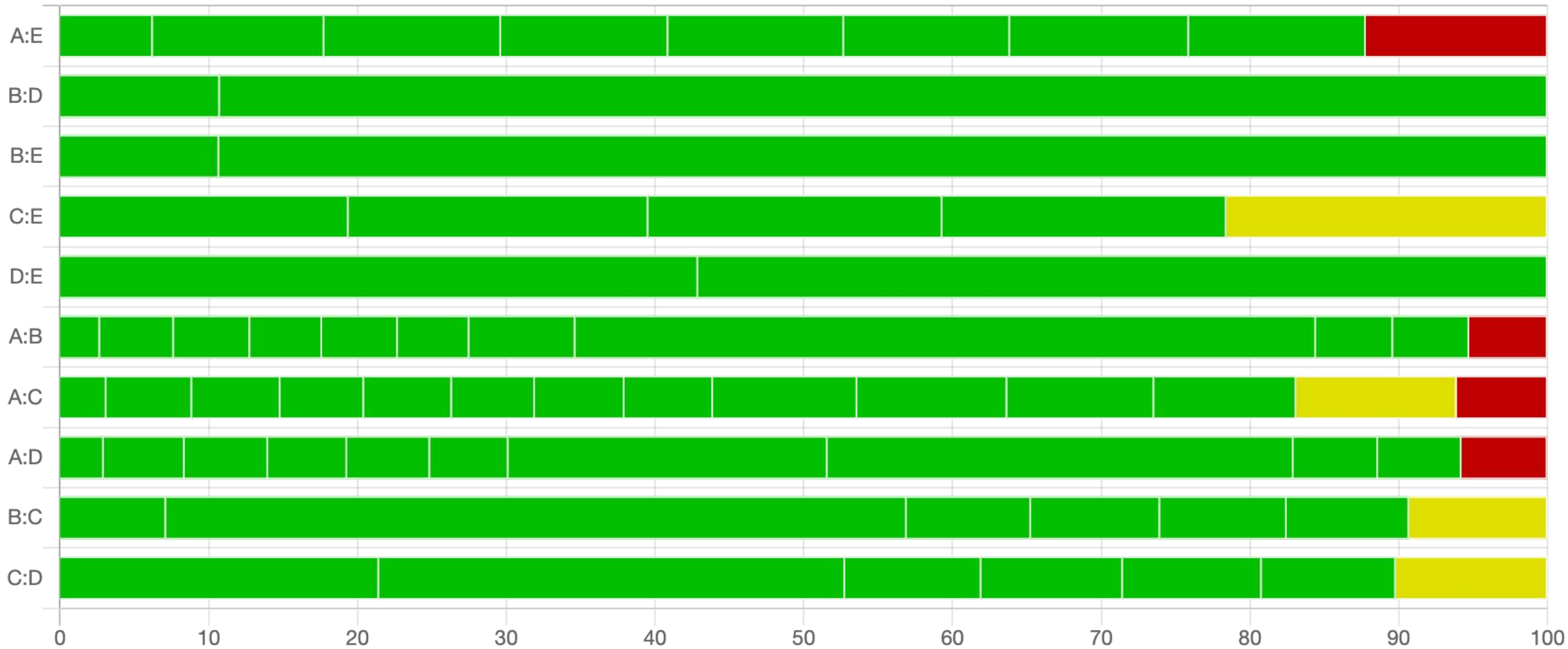

Table S7.1: Transitivity (Indirectness) Assessment

	Characteristics relevant to transitivity assessment							
Intervention	Age (years)	Male/female, n; % male	Training status	EIMD model	Supplementation duration	Supplement timing	Outcome assessment tool	Active component/dose
Beetroot	26.72 ± 9.57	178/38; 82.4% male	Mixed	Mixed lower-limb/sport-specific models	Mostly multi-day	Mixed pre- and/or post-exercise timing	VAS, NRS, and PPT	Beetroot-derived nitrate, with betalains/polyphenols in some trials; nitrate dose was reported as approx. 0.205-8.9 mmol or 125-450 mg/serving where available.
Watermelon	23.58 ± 3.21	26/0; 100% male	Mixed	Lower-limb cycle/squat models	Acute only	Pre-exercise	Likert-type scale	Watermelon-derived L-citrulline; approx. 0.5-1.17 g/serving
Synthetic precursors	26.31 ± 6.77	87/17; 83.7% male	Untrained or recreational	Resistance-based upper-/lower-limb models	Mixed acute and multi-day	Mixed pre- and/or post-exercise timing	VAS and Likert-type scale	Citrulline malate: 6-8 g/serving; L-arginine: 6 g/serving
Watermelon + citrulline	29.28 ± 9.18	60/14; 81.1% male	Mixed	Mixed lower-limb/sport-specific models	Acute only	Pre-exercise	Likert-type scale	Watermelon-based added/enriched L-citrulline; approx. 0.78-6.0 g L-citrulline/serving

Note: Values summarize the distribution of potential effect modifiers across intervention nodes. Age and sex were summarized descriptively using the available study-level or group-level data. Supplementation duration was classified as acute when supplementation was administered as a single dose or within the same day of the exercise bout, and as multi-day when supplementation was repeated over more than one day. Timing was classified as pre-exercise, post-exercise, or pre- and post-exercise. These variables were used to assess clinical similarity and transitivity rather than to perform formal between-node statistical comparisons.

Imprecision: Certainty of evidence for each network estimate was assessed using the CINeMA framework. Imprecision was judged according to whether the 95% confidence interval crossed the prespecified threshold for a clinically important effect, defined as 0.20 SMD on the DOMS scale.

Heterogeneity: Heterogeneity was evaluated by comparing confidence intervals and prediction intervals with the same 0.20 SMD threshold for clinical importance used in the imprecision assessment. Between-study heterogeneity was quantified using tau squared and interpreted descriptively.

Incoherence: In CINeMA, incoherence refers to disagreement between direct and indirect evidence. Local incoherence could only be assessed for comparisons with both direct and indirect evidence. For comparisons without both sources of evidence, local incoherence could not be assessed. The global design-by-treatment interaction test was considered separately and interpreted cautiously because of the sparse, predominantly placebo-centred network and the limited number of comparisons with both direct and indirect evidence.

Table S7.2: CINeMA Results of 48-H DOMS

Comparison	Within-study bias	Reporting bias	Indirectness	Imprecision	Heterogeneity	Incoherence	Confidence rating
Beetroot:Placebo	No concerns	Low risk	No concerns	No concerns	Major concerns	Major concerns	Low
Watermelon:Watermelon + citrulline	No concerns	Low risk	No concerns	Major concerns	No concerns	No concerns	Low
Watermelon:Placebo	No concerns	Low risk	No concerns	Major concerns	No concerns	No concerns	Low
Synthetic precursors:Placebo	No concerns	Low risk	No concerns	Major concerns	No concerns	Major concerns	Low
Watermelon + citrulline:Placebo	No concerns	Low risk	No concerns	Major concerns	No concerns	Major concerns	Low
Beetroot:Watermelon	No concerns	Low risk	No concerns	Major concerns	No concerns	Major concerns	Low
Beetroot:Synthetic precursors	No concerns	Low risk	No concerns	No concerns	Major concerns	Major concerns	Low
Beetroot:Watermelon + citrulline	No concerns	Low risk	No concerns	Major concerns	No concerns	Major concerns	Low
Watermelon:Synthetic precursors	No concerns	Low risk	No concerns	Major concerns	No concerns	Major concerns	Low
Synthetic precursors:Watermelon + citrulline	No concerns	Low risk	No concerns	Major concerns	No concerns	Major concerns	Low

Appendix 8: Funnel plots

Funnel plots were used to explore small-study effects and possible publication bias in the network meta-analysis of nitric oxide precursor supplementation for exercise-induced muscle damage outcomes. Each plot incorporated all direct comparisons between active interventions and placebo. Funnel plot asymmetry was formally examined using Egger's test. A p value greater than 0.05 was interpreted as indicating no significant asymmetry and thus little evidence of substantial publication bias, whereas a "p" value below 0.05 suggested potential small-study effects.

Figure S8.1: DOMS at 48-H Post-Exercise

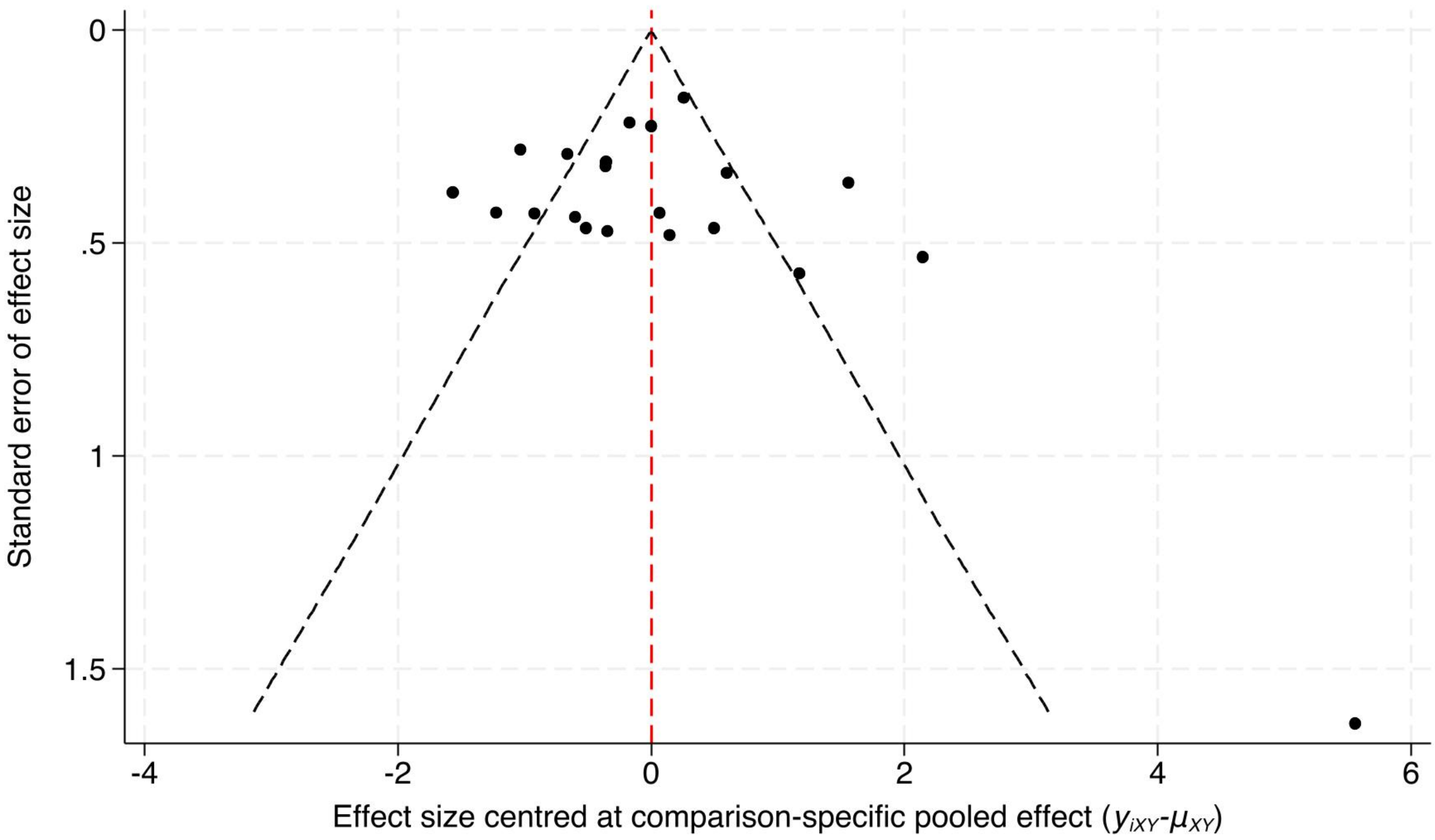

Egger's test: bias coefficient = 2.52 (SE = 1.27), t = 1.98, p = 0.061, 95% CI −0.12 to 5.16

Figure S8.2: DOMS at 24-H Post-Exercise

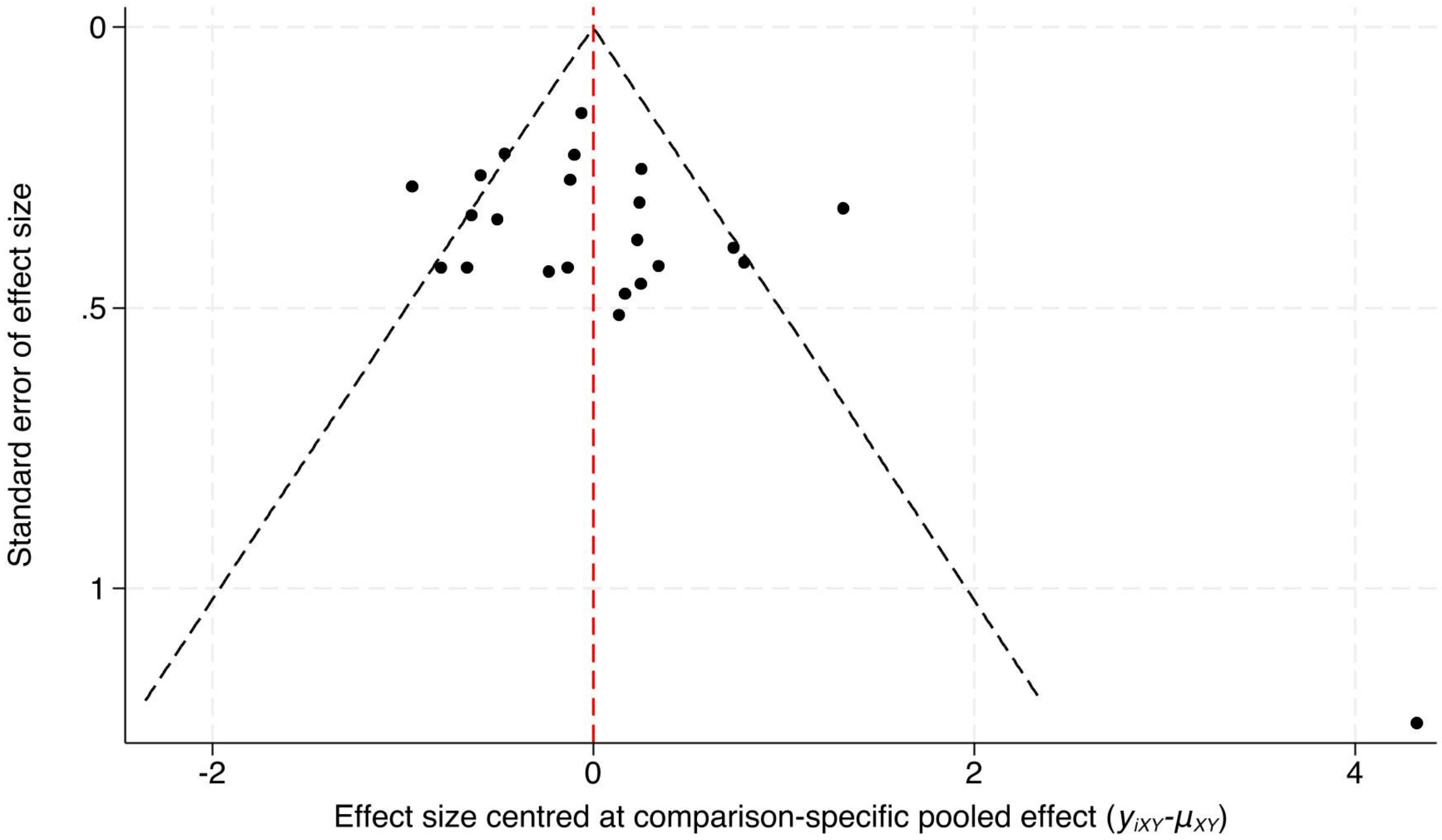

Egger's test: bias coefficient = 2.51 (SE = 1.36), t = 1.69, p = 0.107, 95% CI −0.55 to 5.16

Figure S8.3: DOMS at 72-H Post-Exercise

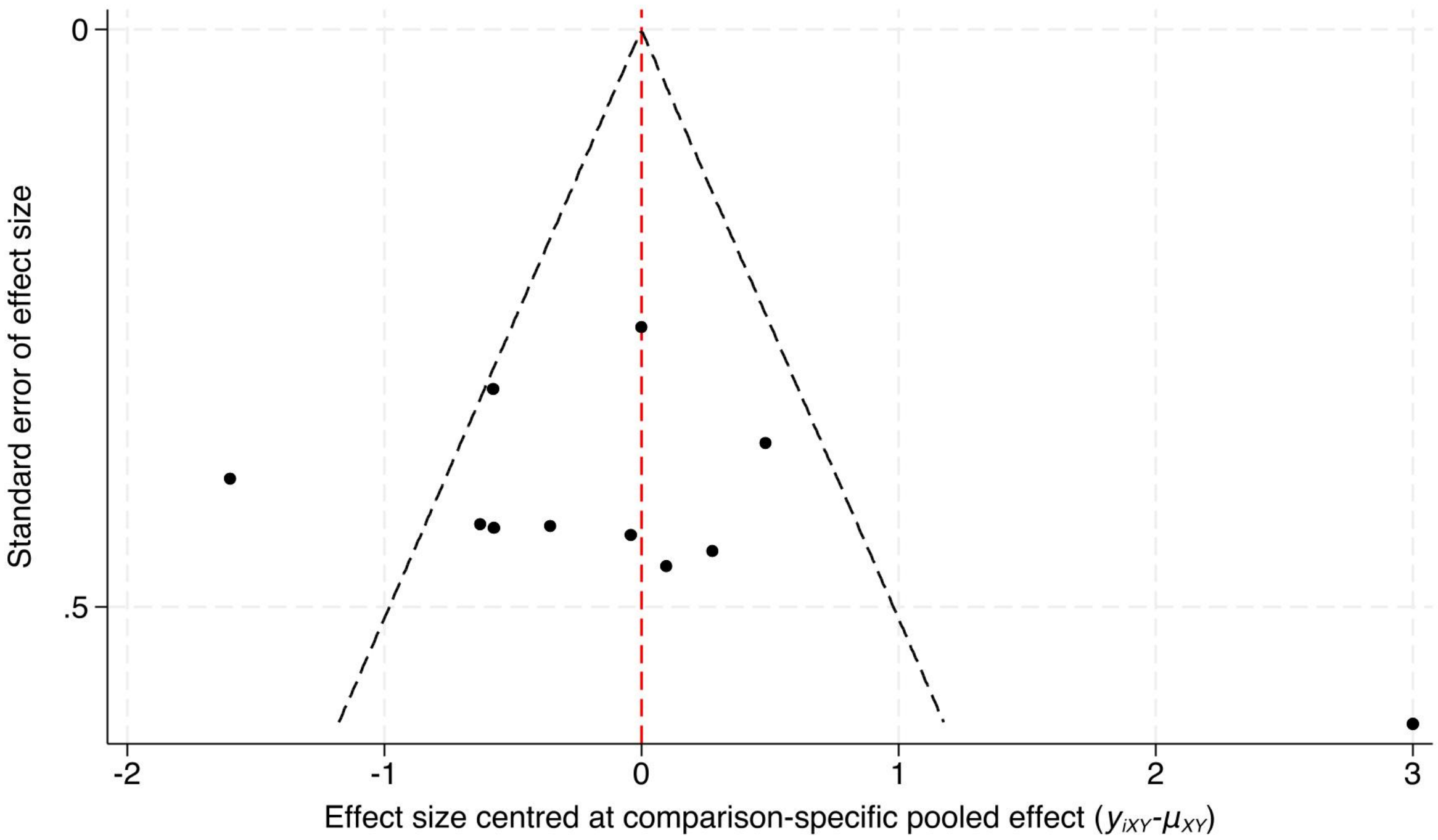

Egger's test: bias coefficient = 2.58 (SE = 3.39), t = 0.76, p = 0.466, 95% CI −5.09 to 10.25

Appendix 9: Sensitivity analyses

Table S9.1: Sensitivity analyses for 48-h DOMS based on study, intervention, and outcome characteristics

**Intervention**	**Main analysis**	**Excluding laboratory-controlled lower-limb mechanical EIMD models**	**Excluding real-world or sport-specific mixed models**	**Excluding mixed-muscle resistance models**	**Excluding atypical or lower-certainty EIMD models**	**Restricted to laboratory-controlled lower-limb mechanical models**	**Excluding PPT-based outcomes**	**Restriction to citrulline malate interventions**	**Restriction to pre-exercise supplementation protocols**	**excluding the powder-based beetroot formulation**
Beetroot-derived nitrate	-1.18 (-1.91, -0.45)	-1.45 (-2.73, -0.17)	-0.80 (-1.65, 0.05)	-1.36 (-2.23, -0.50)	-1.21 (-2.01, -0.42)	-1.01 (-2.29, 0.26)	-1.74 (-2.81, -0.66)	-1.20 (-1.96, -0.43)	-2.75 (-5.35, -0.14)	-1.32 (-2.11, -0.54)
watermelon-derived citrulline	-0.09 (-1.47, 1.28)	-0.08 (-2.63, 2.47)	-0.13 (-1.35, 1.09)	-0.09 (-1.66, 1.47)	-0.08 (-2.20, 2.04)	-0.28 (-2.68, 2.13)	-0.09 (-1.64, 1.45)	-0.09 (-1.54, 1.36)	-0.10 (-2.01, 1.82)	-0.09 (-1.49, 1.30)
synthetic or isolated amino	-0.11 (-1.05, 0.84)	-0.27 (-2.24, 1.70)	-0.11 (-0.92, 0.70)	0.01 (-1.37, 1.40)	-0.10 (-1.14, 0.93)	0.01 (-1.41, 1.44)	-0.10 (-1.16, 0.95)	-0.09 (-1.19, 1.01)	-0.20 (-1.86, 1.46)	-0.10 (-1.06, 0.85)
watermelon plus added citrulline	-0.51 (-1.73, 0.72)	-0.18 (-2.16, 1.80)	-0.60 (-1.92, 0.72)	-0.50 (-1.88, 0.88)	-0.73 (-2.34, 0.89)	-1.13 (-3.58, 1.33)	-0.50 (-1.86, 0.86)	-0.50 (-1.79, 0.78)	-0.50 (-2.17, 1.17)	-0.51 (-1.74, 0.73)

Table S9.2. Sensitivity analyses for 48-h DOMS under alternative within-subject correlation assumptions in crossover trials

Intervention	Main analysis	r = 0.3	r = 0.7	r = 0.9
Beetroot-derived nitrate	-1.18 (-1.91, -0.45)	-1.18 (-1.91, -0.45)	-1.18 (-1.91, -0.45)	-1.18 (-1.91, -0.45)
watermelon-derived citrulline	-0.09 (-1.47, 1.28)	-0.09 (-1.47, 1.28)	-0.09 (-1.47, 1.29)	-0.09 (-1.48, 1.29)
synthetic or isolated amino acid precursors	-0.11 (-1.05, 0.84)	-0.11 (-1.05, 0.84)	-0.10 (-1.05, 0.84)	-0.10 (-1.04, 0.84)
watermelon plus added citrulline	-0.51 (-1.73, 0.72)	-0.51 (-1.73, 0.71)	-0.51 (-1.73, 0.72)	-0.51 (-1.73, 0.72)

Note: The primary analysis assumed a within-subject correlation of r = 0.5 for crossover trials. Sensitivity analyses were repeated using alternative correlation coefficients of r = 0.3, 0.7, and 0.9.

Appendix 10: Subgroup analysis of training status

Subgroup analyses were performed by participants' athletic status (athletes vs non-athletes) and by supplementation duration, classified as acute versus longer-term interventions.

Figure S10.1: Forest plot of DOMS at 48-H Post-Exercise— trained participants and athletes

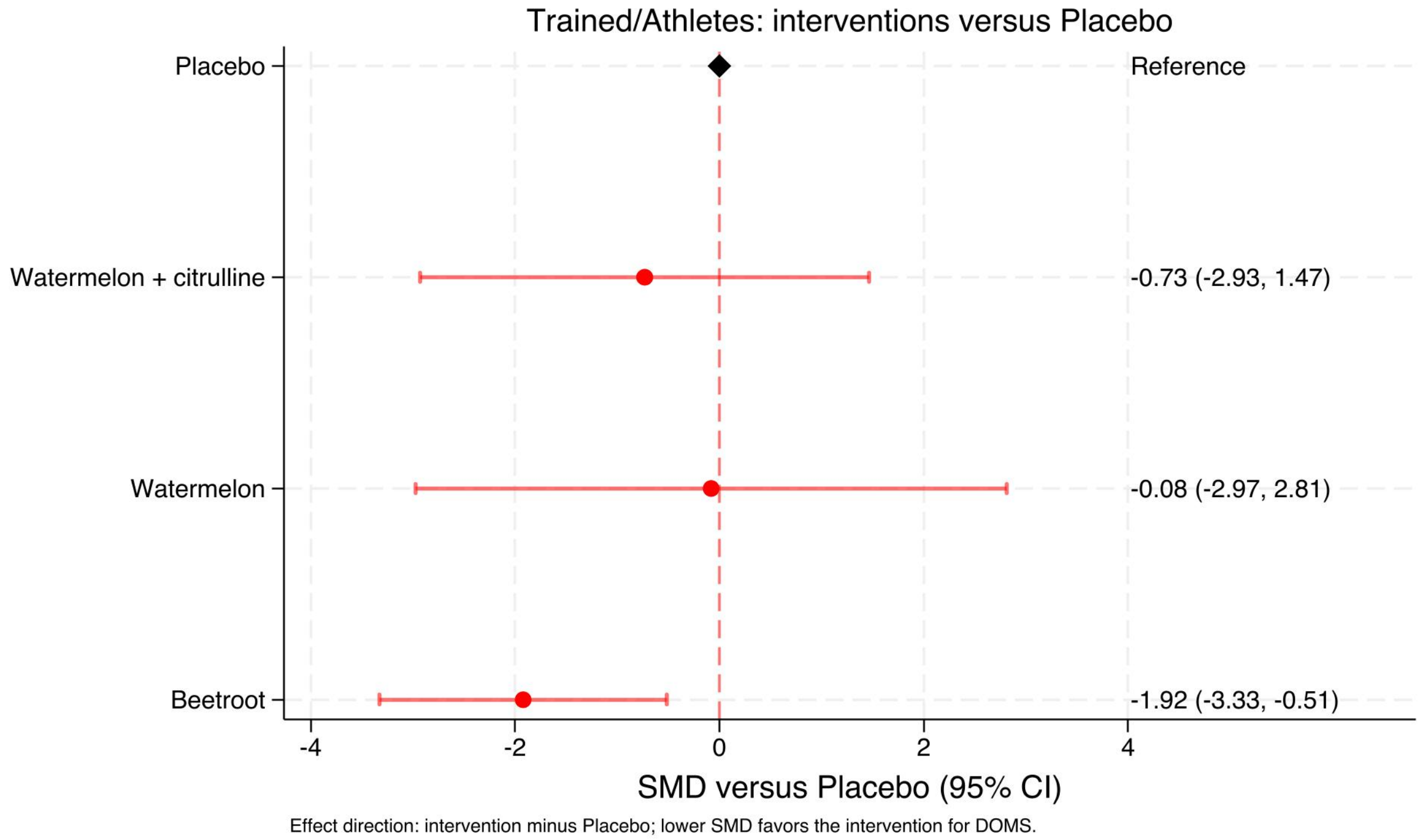

Table S10.1: League table of DOMS at 48-H Post-Exercise— trained participants and athletes

Beetroot			
-1.84 (-5.06, 1.38)	Watermelon		
-1.19 (-3.80, 1.42)	0.65 (-2.26, 3.56)	Watermelon + citrulline	
-1.92 (-3.33, -0.51)	-0.08 (-2.97, 2.81)	-0.73 (-2.93, 1.47)	Placebo

Figure S10.2: Forest plot of DOMS at 48-H Post-Exercise— untrained and recreationally active participants

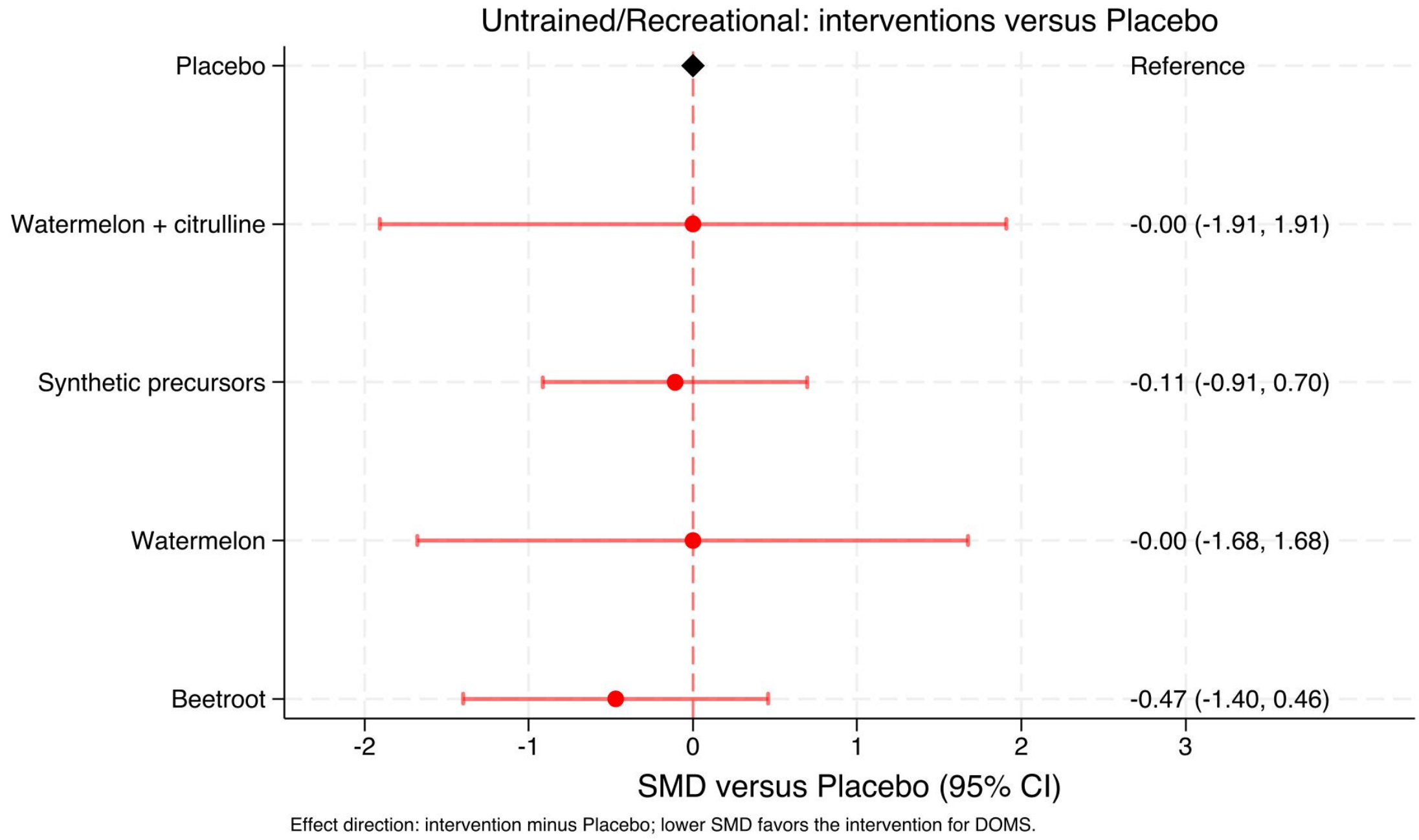

Table S10.2: League table of DOMS at 48-H Post-Exercise— untrained and recreationally active participants

Beetroot				
-0.47 (-2.39, 1.45)	Watermelon			
-0.36 (-1.59, 0.87)	0.11 (-1.75, 1.97)	Synthetic precursors		
-0.47 (-2.59, 1.65)	0.00 (-1.91, 1.91)	-0.11 (-2.18, 1.96)	Watermelon + citrulline	
-0.47 (-1.40, 0.46)	-0.00 (-1.68, 1.68)	-0.11 (-0.91, 0.70)	-0.00 (-1.91, 1.91)	Placebo

Figure S10.3: Forest plot of 48-h DOMS in the acute supplementation subgroup.

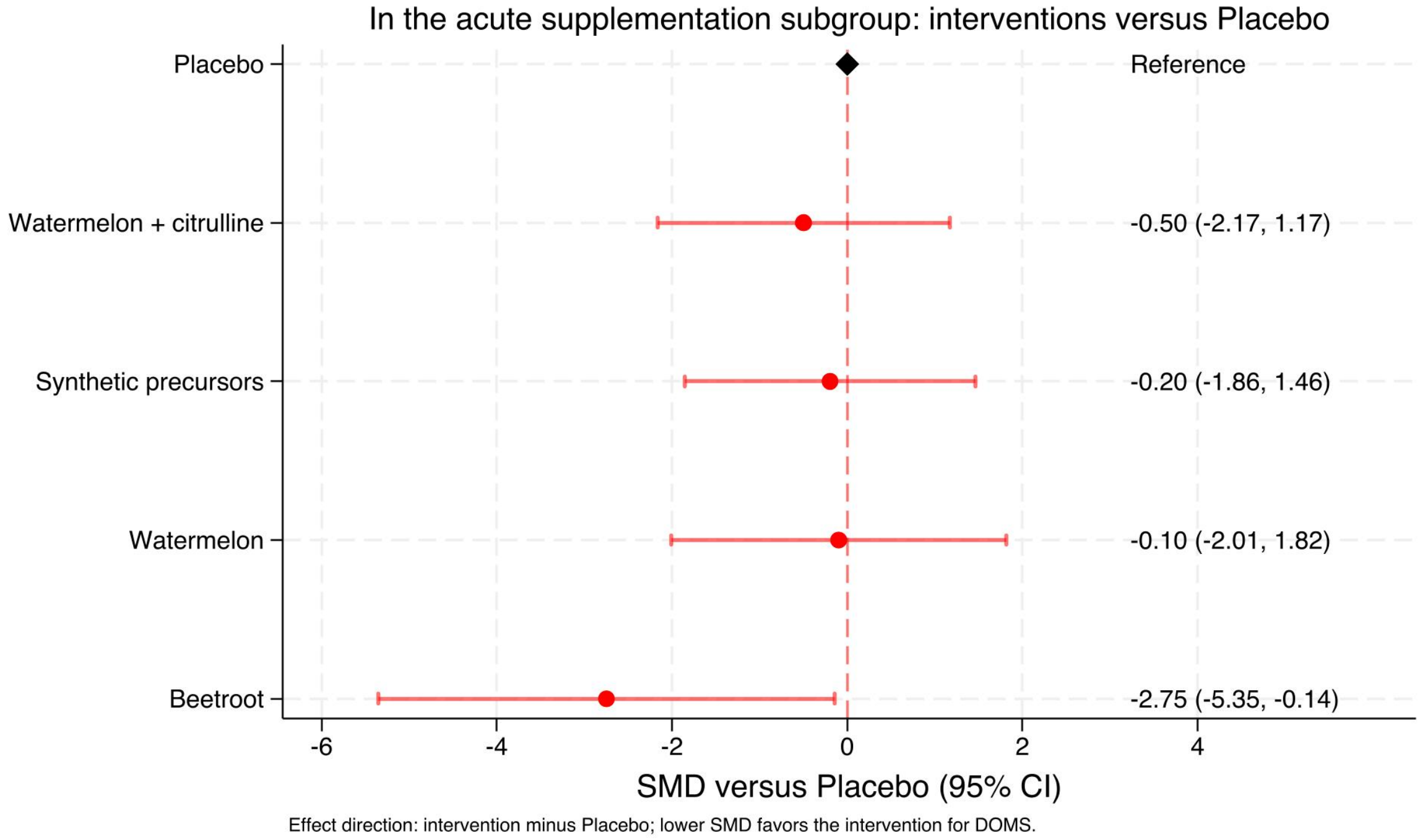

Table S10.3: League table of 48-h DOMS in the acute supplementation subgroup.

Beetroot				
-2.65 (-5.88, 0.58)	Watermelon			
-2.55 (-5.64, 0.54)	0.10 (-2.43, 2.63)	Synthetic precursors		
-2.25 (-5.35, 0.85)	0.40 (-1.55, 2.36)	0.30 (-2.05, 2.65)	Watermelon + citrulline	
-2.75 (-5.35, -0.14)	-0.10 (-2.01, 1.82)	-0.20 (-1.86, 1.46)	-0.50 (-2.17, 1.17)	Placebo

Figure S10.4: Forest plot of 48-h DOMS in the multi-day supplementation subgroup.

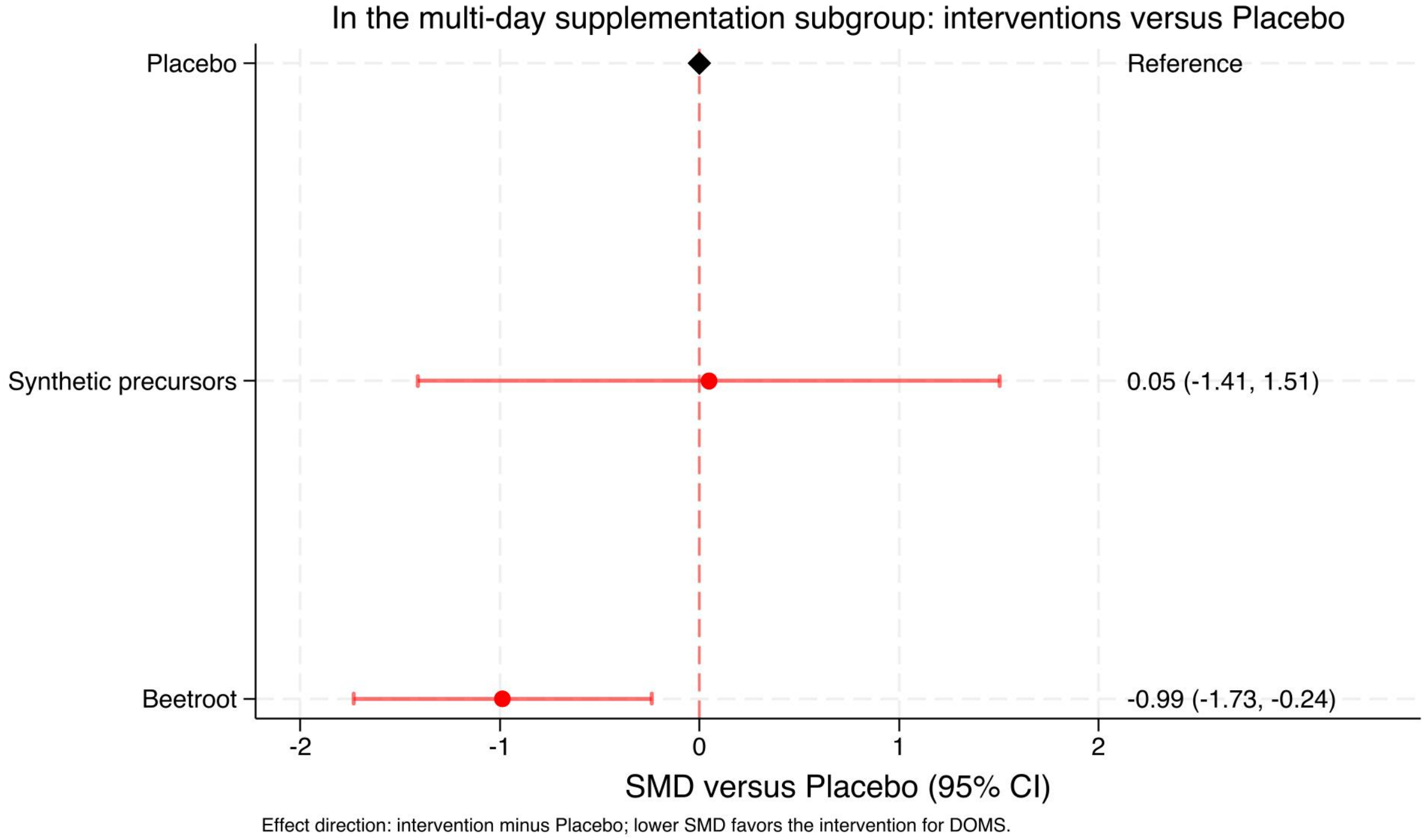

Table S10.4: League table of 48-h DOMS in the multi-day supplementation subgroup.

Beetroot		
-1.04 (-2.67, 0.60)	Synthetic precursors	
-0.99 (-1.73, -0.24)	0.05 (-1.41, 1.51)	Placebo

Appendix 11: PRISMA checklist

Table S11.1: PRISMA 2020 checklist

Section and Topic	Item #	Checklist item	Location where item is reported
**TITLE**			
Title	1	Identify the report as a systematic review.	Manuscript p. 1
**ABSTRACT**			
Abstract	2	See the PRISMA 2020 for Abstracts checklist.	Manuscript pp. 1-2
**INTRODUCTION**			
Rationale	3	Describe the rationale for the review in the context of existing knowledge.	Manuscript pp. 2-3
Objectives	4	Provide an explicit statement of the objective(s) or question(s) the review addresses.	Manuscript p. 3
**METHODS**			
Eligibility criteria	5	Specify the inclusion and exclusion criteria for the review and how studies were grouped for the syntheses.	Manuscript pp. 3-4; Supplementary file pp. 6-9 (Tables S2.1-S2.2)
Information sources	6	Specify all databases, registers, websites, organisations, reference lists and other sources searched or consulted to identify studies. Specify the date when each source was last searched or consulted.	Manuscript p. 3; Supplementary file pp. 2-5
Search strategy	7	Present the full search strategies for all databases, registers and websites, including any filters and limits used.	Supplementary file pp. 2-5 (Tables S1-S4)
Selection process	8	Specify the methods used to decide whether a study met the inclusion criteria of the review, including how many reviewers screened each record and each report retrieved, whether they worked independently, and if applicable, details of automation tools used in the process.	Manuscript p. 4; Figure 1 on p. 7
Data collection process	9	Specify the methods used to collect data from reports, including how many reviewers collected data from each report, whether they worked independently, any processes for obtaining or confirming data from study investigators, and if applicable, details of automation tools used in the process.	Manuscript pp. 4-5; Supplementary file p. 6 (Table S2.1)
Data items	10a	List and define all outcomes for which data were sought. Specify whether all results that were compatible with each outcome domain in each study were sought (e.g. for all measures, time points, analyses), and if not, the methods used to decide which results to collect.	Manuscript p. 5; Supplementary file p. 6 (Table S2.1)
	10b	List and define all other variables for which data were sought (e.g. participant and intervention characteristics, funding sources). Describe any assumptions made about any missing or unclear information.	Manuscript pp. 4-5; Supplementary file pp. 6-9 (Tables S2.1-S2.2)
Study risk of bias assessment	11	Specify the methods used to assess risk of bias in the included studies, including details of the tool(s) used, how many reviewers assessed each study and whether they worked independently, and if applicable, details of automation tools used in the process.	Manuscript p. 5; Supplementary file p. 10 (Table S3)
Effect measures	12	Specify for each outcome the effect measure(s) (e.g. risk ratio, mean difference) used in the synthesis or presentation of results.	Manuscript pp. 5-6
Synthesis methods	13a	Describe the processes used to decide which studies were eligible for each synthesis (e.g. tabulating the study intervention characteristics and comparing against the planned groups for each synthesis (item #5)).	Manuscript pp. 3-4, 6; Supplementary file pp. 7-9 (Table S2.2)
	13b	Describe any methods required to prepare the data for presentation or synthesis, such as handling of missing summary statistics, or data conversions.	Manuscript pp. 5-6
	13c	Describe any methods used to tabulate or visually display results of individual studies and syntheses.	Manuscript p. 6; Supplementary file pp. 11-19
	13d	Describe any methods used to synthesize results and provide a rationale for the choice(s). If meta-analysis was performed, describe the model(s), method(s) to identify the presence and extent of statistical heterogeneity, and software package(s) used.	Manuscript pp. 5-6; Supplementary

Section and Topic	Item #	Checklist item	Location where item is reported
			file pp. 11, 15-19
	13e	Describe any methods used to explore possible causes of heterogeneity among study results (e.g. subgroup analysis, meta-regression).	Manuscript p. 6; Supplementary file pp. 20-21, 24-28
	13f	Describe any sensitivity analyses conducted to assess robustness of the synthesized results.	Manuscript p. 6; Supplementary file pp. 27-28
Reporting bias assessment	14	Describe any methods used to assess risk of bias due to missing results in a synthesis (arising from reporting biases).	Manuscript p. 5; Supplementary file pp. 25-26
Certainty assessment	15	Describe any methods used to assess certainty (or confidence) in the body of evidence for an outcome.	Manuscript p. 5; Supplementary file pp. 20-24
**RESULTS**			
Study selection	16a	Describe the results of the search and selection process, from the number of records identified in the search to the number of studies included in the review, ideally using a flow diagram.	Manuscript p. 6; Figure 1 on p. 7
	16b	Cite studies that might appear to meet the inclusion criteria, but which were excluded, and explain why they were excluded.	Manuscript p. 7; Figure 1 on p. 7
Study characteristics	17	Cite each included study and present its characteristics.	Manuscript pp. 8-11; Supplementary file pp. 7-9 (Table S2.2)
Risk of bias in studies	18	Present assessments of risk of bias for each included study.	Manuscript pp. 11-12; Supplementary file p. 10 (Table S3)
Results of individual studies	19	For all outcomes, present, for each study: (a) summary statistics for each group (where appropriate) and (b) an effect estimate and its precision (e.g. confidence/credible interval), ideally using structured tables or plots.	Manuscript pp. 12-15; Supplementary file pp. 14-19, 27-32
Results of syntheses	20a	For each synthesis, briefly summarise the characteristics and risk of bias among contributing studies.	Manuscript pp. 11-15; Supplementary file pp. 10, 20-24
	20b	Present results of all statistical syntheses conducted. If meta-analysis was done, present for each the summary estimate and its precision (e.g. confidence/credible interval) and measures of statistical heterogeneity. If comparing groups, describe the direction of the effect.	Manuscript pp. 12-15; Supplementary file pp. 11-19
	20c	Present results of all investigations of possible causes of heterogeneity among study results.	Manuscript pp. 14-15; Supplementary file pp. 20-24, 27-32
	20d	Present results of all sensitivity analyses conducted to assess the robustness of the synthesized results.	Manuscript p. 15; Supplementary file p. 27-28
Reporting biases	21	Present assessments of risk of bias due to missing results (arising from reporting biases) for each synthesis assessed.	Manuscript p. 11; Supplementary file pp. 25-26
Certainty of evidence	22	Present assessments of certainty (or confidence) in the body of evidence for each outcome assessed.	Manuscript pp. 11-12; Supplementary file pp. 20-24
**DISCUSSION**			
Discussion	23a	Provide a general interpretation of the results in the context of other evidence.	Manuscript pp. 15-18
	23b	Discuss any limitations of the evidence included in the review.	Manuscript pp. 17-18
	23c	Discuss any limitations of the review processes used.	Manuscript pp. 17-18

Section and Topic	Item #	Checklist item	Location where item is reported
	23d	Discuss implications of the results for practice, policy, and future research.	Manuscript p. 18
OTHER INFORMATION			
Registration and protocol	24a	Provide registration information for the review, including register name and registration number, or state that the review was not registered.	Manuscript p. 3
	24b	Indicate where the review protocol can be accessed, or state that a protocol was not prepared.	Manuscript p. 3 (PROSPERO)
	24c	Describe and explain any amendments to information provided at registration or in the protocol.	Not applicable; no protocol amendments reported
Support	25	Describe sources of financial or non-financial support for the review, and the role of the funders or sponsors in the review.	Manuscript p. 19
Competing interests	26	Declare any competing interests of review authors.	Manuscript p. 19
Availability of data, code and other materials	27	Report which of the following are publicly available and where they can be found: template data collection forms; data extracted from included studies; data used for all analyses; analytic code; any other materials used in the review.	Manuscript p. 19; Supplementary file pp. 2-32

Table S11.2: PRISMA-NMA checklist

Section/Topic	Item #	Checklist Item	Reported on Page #
**TITLE**			
Title	1	Identify the report as a systematic review *incorporating a network meta-analysis (or related form of meta-analysis).*	Manuscript p. 1
**ABSTRACT**			
Structured summary	2	Provide a structured summary including, as applicable:<br>**Background:** main objectives<br>**Methods:** data sources; study eligibility criteria, participants, and interventions; study appraisal; and *synthesis methods, such as network meta-analysis.*<br>**Results:** number of studies and participants identified; summary estimates with corresponding confidence/credible intervals; *treatment rankings may also be discussed. Authors may choose to summarize pairwise comparisons against a chosen treatment included in their analyses for brevity.*<br>**Discussion/Conclusions:** limitations; conclusions and implications of findings.<br>**Other:** primary source of funding; systematic review registration number with registry name.	Manuscript p. 1-2
**INTRODUCTION**			
Rationale	3	Describe the rationale for the review in the context of what is already known*, including mention of why a network meta-analysis has been conducted.*	Manuscript pp. 2-3
Objectives	4	Provide an explicit statement of questions being addressed, with reference to participants, interventions, comparisons, outcomes, and study design (PICOS).	Manuscript p. 3
**METHODS**			
Protocol and registration	5	Indicate whether a review protocol exists and if and where it can be accessed (e.g., Web address); and, if available, provide registration information, including registration number.	Manuscript p. 3
Eligibility criteria	6	Specify study characteristics (e.g., PICOS, length of follow-up) and report characteristics (e.g., years considered, language, publication status) used as criteria for eligibility, giving rationale. *Clearly describe eligible treatments included in the treatment network, and note whether any have been clustered or merged into the same node (with justification).*	Manuscript pp. 3-4; Supplementary file pp. 6-9
Information sources	7	Describe all information sources (e.g., databases with dates of coverage, contact with study authors to identify additional studies) in the search and date last	Manuscript p. 3; Supplementary file pp. 2-5

		searched.	
Search	8	Present full electronic search strategy for at least one database, including any limits used, such that it could be repeated.	Supplementary file pp. 2-5 (Tables S1-S4)
Study selection	9	State the process for selecting studies (i.e., screening, eligibility, included in systematic review, and, if applicable, included in the meta-analysis).	Manuscript p. 4; Figure 1 on p. 7
Data collection process	10	Describe method of data extraction from reports (e.g., piloted forms, independently, in duplicate) and any processes for obtaining and confirming data from investigators.	Manuscript pp. 4-5; Supplementary file p. 6
Data items	11	List and define all variables for which data were sought (e.g., PICOS, funding sources) and any assumptions and simplifications made.	Manuscript pp. 4-5; Supplementary file pp. 6-9
**Geometry of the network**	**S1**	Describe methods used to explore the geometry of the treatment network under study and potential biases related to it. This should include how the evidence base has been graphically summarized for presentation, and what characteristics were compiled and used to describe the evidence base to readers.	Manuscript p. 6; Figures 3-4 on pp. 12-13
Risk of bias within individual studies	12	Describe methods used for assessing risk of bias of individual studies (including specification of whether this was done at the study or outcome level), and how this information is to be used in any data synthesis.	Manuscript p. 5; Supplementary file p. 10
Summary measures	13	State the principal summary measures (e.g., risk ratio, difference in means). *Also describe the use of additional summary measures assessed, such as treatment rankings and surface under the cumulative ranking curve (SUCRA) values, as well as modified approaches used to present summary findings from meta-analyses.*	Manuscript pp. 5-6; Supplementary file pp. 14-19
Planned methods of analysis	14	Describe the methods of handling data and combining results of studies for each network meta-analysis. This should include, but not be limited to:<br>• *Handling of multi-arm trials;*<br>• *Selection of variance structure;*<br>• *Selection of prior distributions in Bayesian analyses; and*<br>• *Assessment of model fit.*	Manuscript pp. 5-6
**Assessment of Inconsistency**	**S2**	Describe the statistical methods used to evaluate the agreement of direct and indirect evidence in the treatment network(s) studied. Describe efforts taken to address its presence when found.	Manuscript p. 6; Supplementary file pp. 11-13, 20-24
Risk of bias across studies	15	Specify any assessment of risk of bias that may affect the cumulative evidence (e.g., publication bias, selective reporting within studies).	Manuscript p. 5; Supplementary file pp. 25-26
Additional analyses	16	Describe methods of additional analyses if done, indicating which were pre-specified. This may include, but not be limited to, the following:<br>• Sensitivity or subgroup analyses;<br>• Meta-regression analyses;<br>• *Alternative formulations of the treatment network; and*	Manuscript p. 6; Supplementary file pp. 27-32

		• *Use of alternative prior distributions for Bayesian analyses (if applicable).*	
**RESULTS**			
Study selection	17	Give numbers of studies screened, assessed for eligibility, and included in the review, with reasons for exclusions at each stage, ideally with a flow diagram.	Manuscript p. 6; Figure 1 on p. 7
**Presentation of network structure**	**S3**	Provide a network graph of the included studies to enable visualization of the geometry of the treatment network.	Manuscript pp. 12-13 (Figures 3-4)
**Summary of network geometry**	**S4**	Provide a brief overview of characteristics of the treatment network. This may include commentary on the abundance of trials and randomized patients for the different interventions and pairwise comparisons in the network, gaps of evidence in the treatment network, and potential biases reflected by the network structure.	Manuscript pp. 12-13
Study characteristics	18	For each study, present characteristics for which data were extracted (e.g., study size, PICOS, follow-up period) and provide the citations.	Manuscript pp. 8-11; Supplementary file pp. 7-9
Risk of bias within studies	19	Present data on risk of bias of each study and, if available, any outcome level assessment.	Manuscript pp. 11-12; Supplementary file p. 10
Results of individual studies	20	For all outcomes considered (benefits or harms), present, for each study: 1) simple summary data for each intervention group, and 2) effect estimates and confidence intervals. *Modified approaches may be needed to deal with information from larger networks.*	Manuscript pp. 12-15; Supplementary file pp. 14-19, 27-32
Synthesis of results	21	Present results of each meta-analysis done, including confidence/credible intervals. *In larger networks, authors may focus on comparisons versus a particular comparator (e.g. placebo or standard care), with full findings presented in an appendix. League tables and forest plots may be considered to summarize pairwise comparisons.* If additional summary measures were explored (such as treatment rankings), these should also be presented.	Manuscript pp. 12-15; Supplementary file pp. 12-17
**Exploration for inconsistency**	**S5**	Describe results from investigations of inconsistency. This may include such information as measures of model fit to compare consistency and inconsistency models, *P* values from statistical tests, or summary of inconsistency estimates from different parts of the treatment network.	Manuscript p. 11; Supplementary file pp. 11-13, 25-26
Risk of bias across studies	22	Present results of any assessment of risk of bias across studies for the evidence base being studied.	Manuscript p. 11; Supplementary file pp. 22-23
Results of additional analyses	23	Give results of additional analyses, if done (e.g., sensitivity or subgroup analyses, meta-regression analyses*, alternative network geometries studied, alternative choice of prior distributions for Bayesian analyses,* and so forth).	Manuscript pp. 14-15; Supplementary file pp. 27-32

**DISCUSSION**			
Summary of evidence	24	Summarize the main findings, including the strength of evidence for each main outcome; consider their relevance to key groups (e.g., healthcare providers, users, and policy-makers).	Manuscript pp. 15-18
Limitations	25	Discuss limitations at study and outcome level (e.g., risk of bias), and at review level (e.g., incomplete retrieval of identified research, reporting bias). *Comment on the validity of the assumptions, such as transitivity and consistency. Comment on any concerns regarding network geometry (e.g., avoidance of certain comparisons).*	Manuscript pp. 17-18
Conclusions	26	Provide a general interpretation of the results in the context of other evidence, and implications for future research.	Manuscript p. 18
**FUNDING**			
Funding	27	Describe sources of funding for the systematic review and other support (e.g., supply of data); role of funders for the systematic review. This should also include information regarding whether funding has been received from manufacturers of treatments in the network and/or whether some of the authors are content experts with professional conflicts of interest that could affect use of treatments in the network.	Manuscript p. 19
